# Direct imaging with multidimensional labelling and high‐content analysis allows quantitative categorization and characterizations of individual small extracellular vesicles and nanoparticles (sEVPs)

**DOI:** 10.1002/jev2.12520

**Published:** 2024-12-12

**Authors:** Simou Sun, Sarah J. Cox‐Vázquez, Nam‐Joon Cho, Guillermo C. Bazan, Jay T. Groves

**Affiliations:** ^1^ Institute for Digital Molecular Analytics and Science Nanyang Technological University Singapore Singapore; ^2^ Department of Chemistry National University of Singapore Singapore Singapore; ^3^ Institute for Functional Intelligent Materials National University of Singapore Singapore Singapore; ^4^ School of Materials Science and Engineering Nanyang Technological University Singapore Singapore; ^5^ Department of Chemistry University of California Berkeley California USA; ^6^ Current address: Department of Chemistry Stony Brook University New York United States

**Keywords:** Single‐EV Characterizations, High‐Throughput Arrays, Quantitative Imaging, High‐Content Analysis

## Abstract

Small extracellular vesicles and nanoparticles (sEVPs) are cell‐secreted entities with potential as diagnostic biomarkers and therapeutic vehicles. However, significant intrinsic sEVP heterogeneity impedes analysis and understanding of their composition and functions. We employ multidimensional fluorescent labelling on sEVPs, leveraging the robustness of a newly developed membrane probe—conjugated oligoelectrolytes (COEs), and conduct total internal reflection fluorescence (TIRF) microscopy on sEVP arrays. These arrays comprise single sEVPs anchored to a soft material functionalized surface with little bias. We then develop an enhanced algorithm for colocalization analysis of the multiple labels on individual sEVPs and perform deep profiling of particle content. We categorize sEVPs derived from the same cell type into seven distinct subpopulations—some vesicular whereas others non‐vesicular, and we demonstrate that sEVPs from four cell types exhibit quantitatively distinguishable subpopulation distributions. Furthermore, we gain insights into specific particle features within each subpopulation, including CD63 counts, relative particle size, relative concentration of cargoes, and correlations among different cargoes. This high‐content analysis reveals common cargo sorting features in sEVP subpopulations across different cell types and suggests new statistics within the sEVP inherent heterogeneity that could differentiate sEVPs from two types of cancer cells and two types of normal cells. Collectively, our study presents a robust single‐sEVP characterization platform, combining high‐content imaging with comprehensive analysis. This platform is poised to advance sEVP‐based theranostic assays and facilitate exploration into disease‐associated sEVP biogenesis and sEVP‐mediated intercellular communication.

## 1 INTRODUCTION

Cells release extracellular vesicles (EVs) and non‐vesicular extracellular nanoparticles (NPs) of varying sizes and intracellular origins in both physiological and pathological conditions (Jeppesen et al., 2023; Kalluri & LeBleu, 2020). Among the diverse subtypes of EVs, small extracellular vesicles (sEVs), ranging in size from 30 nm to 200 nm, are prevalent in most human biological fluids (Zhang et al., 2019). While the biogenesis of the NPs remains elusive, they share a similar size range with sEVs (Jeppesen et al., 2023; Jeppesen et al., 2019). Separating these two particle groups using routine isolation and purification protocols has proven technically challenging (Zhang et al., 2023; Zhang et al., 2018). Therefore, we collectively refer to sEVs and NPs as small extracellular vesicles and nanoparticles (sEVPs). The last decade has witnessed a surge in interest in sEVPs, primarily due to the following factors: First, sEVPs carry a variety of disease‐related biomarkers, including proteins, RNAs and metabolites, making them an appealing tool for liquid biopsy (Zhou et al., 2020); Second, sEVPs can be engineered to aid tissue targeting and can cross certain biological barriers, such as the blood–brain barrier, positioning them as promising carriers for next‐generation drug delivery (Herrmann et al., 2021); Third, sEVPs have important roles in intercellular communications within many pathological processes, rendering them potential target for novel therapeutic assays (Vatter et al., 2021; Xu et al., 2018). In fact, more than 300 ongoing clinical trials based on sEVPs are currently recorded (https://clinicaltrials.gov/). Nonetheless, it is well acknowledged that sEVPs exhibit substantial inherent heterogeneity arising from factors such as cell type, cargo sorting mechanism, and cellular state, which can significantly impede the specificity and sensitivity of any sEVP‐based theranostic assays (Kalluri & LeBleu, 2020; Van Niel et al., 2022). Hence, a robust workflow for separating distinct particle subpopulations and a high‐throughput assay for quantitatively characterizing individual sEVPs within each subpopulation have long been sought after.

Various methods have been employed to achieve single‐sEVP level measurements. For example, atomic force microscopy was utilized to assess the mechanical properties of single EVPs (Cavallaro et al., 2021). Transmission electron microscopy was employed to characterize morphological features of individual sEVPs (Jung & Mun, 2018). Furthermore, an imaging assay based on Raman spectroscopy was applied to examine the molecular signatures of single EVPs (Kruglik et al., 2019). However, these techniques are constrained by their lack of high‐throughput capabilities. Recent advancements in imaging flow cytometry (IFCM) have enabled sEVP characterizations close to the single‐particle level. Nevertheless, this method still faces technical limitations such as a sensitivity‐based size limit of currently around 40 to 200 nm (Woud et al., 2022). Surface capture followed by fluorescence or scattering imaging has also been discussed in the literature (Silva et al., 2021). However, many studies rely on antibody‐based captures that could lead to detection bias. More recently, it was found that sEVPs can be directly plated onto glass slides, where the particle heterogeneity is preserved (Ferguson et al., 2022; Schürz et al., 2022). However, this setup could introduce perturbations in particle features due to the particle‐glass interaction, and the bare glass surface could lead to significant nonspecific signals. Additionally, a NeutrAvidin (NeuA)‐decorated surface was demonstrated to capture EVs with little bias, but the EVs need to be biotinylated and the surface functionalization process requires multiple steps (Han et al., 2021). Furthermore, due to a current lack of robust water‐soluble dye for lipid membranes, fluorescence imaging of sEVPs stained with membrane probes may suffer from substantial false signals (Melling et al., 2022). Multiplexed fluorescent labelling on a variety of sEVP surface proteins allows some degree of subpopulation categorization and even the identification of cancerous sEVPs. However, this approach requires a library of protein markers (Spitzberg et al., 2023). Moreover, due to the potential spectral cross‐talk among diverse fluorescent markers, the signal intensity in a multiplexed imaging setup often fails to provide precise quantitative information. Imaging‐based technologies that do not rely on fluorescence have also been developed to investigate individual EVs; examples include single‐particle interferometric reflectance imaging sensing (SP‐IRIS) (Daaboul et al., 2016; Deng et al., 2022). Nonetheless, the fabrication process of the imaging chips is complicated.

**FIGURE 1:**
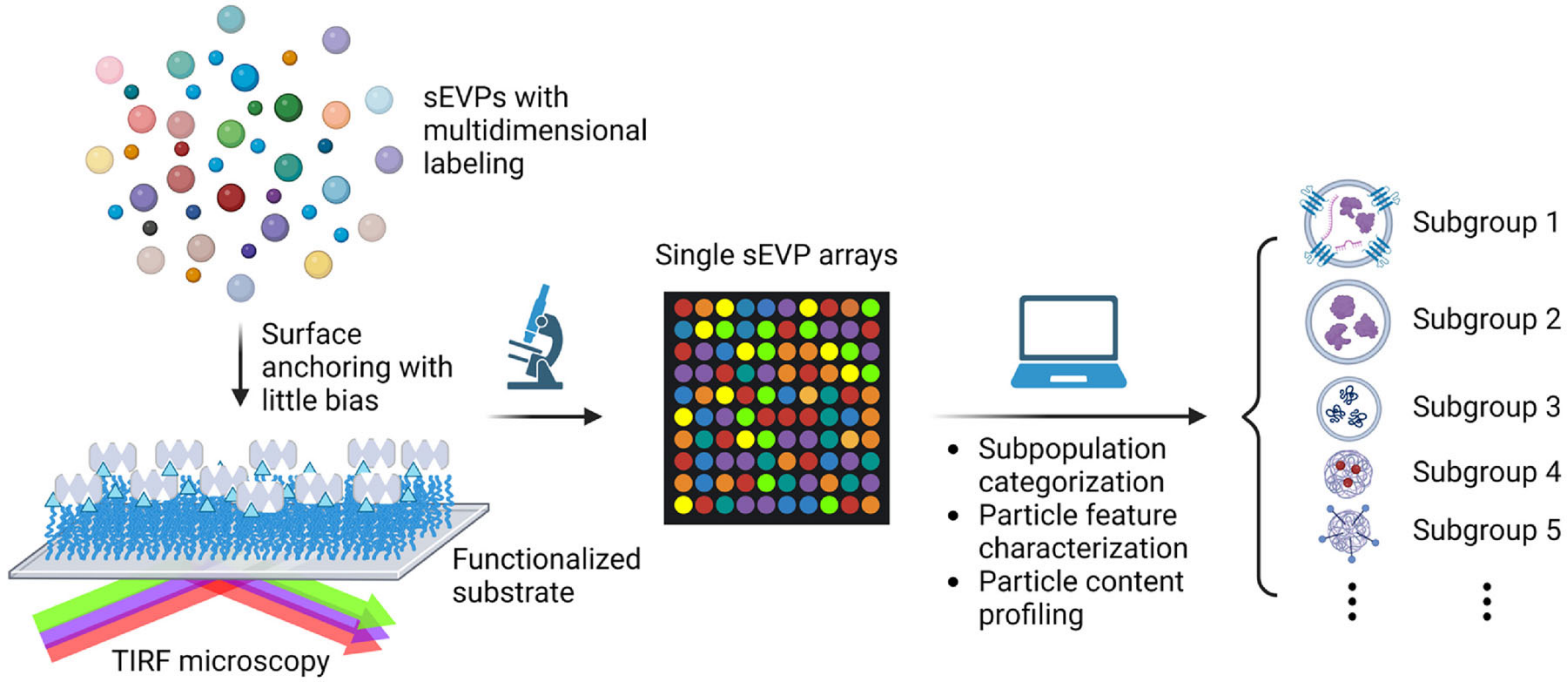
Schematic illustration of the workflow for performing high‐throughput imaging, quantitative subpopulation categorization, and in‐depth analysis of particle features and content at the single‐sEVP level.

Herein, we develop a multidimensional high‐content imaging assay for sEVPs using total internal reflection fluorescence microscopy (TIRFM), where each particle is visualized as a diffraction‐limited bright spot (Figure 1). To reconstruct the three‐dimensional feature of sEVPs, we employ a newly developed water‐soluble lipid membrane probe named conjugated oligoelectrolytes, or COE, in combination with a luminal dye and a surface dye. Moreover, we use an easily fabricated soft material‐functionalized surface to nonspecifically capture sEVPs in a manner with little bias and present them as high‐density arrays. We subsequently establish an analysis workflow that batch processes sEVP images, categorizes the particles into distinct subpopulations, and quantitatively characterizes and profiles individual particles in each subpopulation. We directly separate and identify seven subpopulations within sEVPs from a single cell type and demonstrate comparable populations of vesicles and non‐vesicular NPs in the sEVP sample. Moreover, we unveil distinctive subpopulation distributions across four different cell types. Additionally, an in‐depth analysis of signal intensities from the three fluorescent labels on individual sEVPs allows for a comprehensive profiling of particle content and features within each subpopulation, including parameters such as CD63 counts, relative particle size, relative concentration of cargoes, and correlation among different cargoes. This comprehensive analysis reveals shared cargo sorting characteristics in sEVPs originating from different cell types and suggests novel statistical metrics within the sEVP intrinsic heterogeneity that enables discrimination between two types of cancer cells and two types of normal cells. Collectively, our study presents a robust and high‐throughput platform for direct and quantitative characterizations of sEVPs at the single‐particle level. Our assay allows us to decode precise molecular and population‐level details within the vast heterogeneity of sEVPs. This approach should directly facilitate exploration into disease‐associated sEVP biogenesis and functions, as well as development of sEVP‐based theranostics.

## 2 METHODS

### 2.1 Chemicals

1,2‐dioleoyl‐*sn*‐glycero‐3‐phosphocholine (DOPC), 1,2‐dioleoyl‐*sn*‐glycero‐3‐phospho‐(1′‐rac‐glycerol) (DOPG). 1,2‐distearoyl‐*sn*‐glycero‐3‐phosphoethanolamine‐N‐[biotinyl(polyethylene glycol)‐2000] (DSPE‐PEG(2000) biotin), 1,2‐dioleoyl‐*sn*‐glycero‐3‐phosphoethanolamine‐N‐(lissamine rhodamine B sulfonyl) (Rhodamine B‐DOPE), and 1,2‐dioleoyl‐*sn*‐glycero‐3‐phosphoethanolamine‐N‐(Cyanine 5) (Cy5‐DOPE) were purchased from Avanti Polar Lipids. PLL(20)‐g[3.5]‐ PEG(2)/PEG(3.4)‐ biotin(50%) was purchased from SuSoS. NeutrAvidin (NeuA) protein was purchased from Life Technologies. Lyophilized PC‐3 (human metastatic prostate cancer cell line) exosome standards were purchased from Abcam. Phosphate buffer saline (PBS) was purchased from Corning. CellVue Claret and PKH26 were purchased from Sigma‐Aldrich. CellTrace CFSE (carboxyfluorescein succinimidyl ester) cell proliferation kit was purchased from Life Technologies. PE/Cyanine5 anti‐human CD63 antibody (clone: H5C6, reactivity verified) was purchased from Biolegend. COE‐Ben was synthesized and characterized following the published protocol (Zhou et al., 2023). High density lipoprotein (HDL) from human plasma (quality level: 200) was purchased from Sigma‐Aldrich. Proteinase K (recombinant, PCR grade) was purchased from Life Technologies.

### 2.2 Synthesis and staining of small unilamellar vesicles

Small unilamellar vesicles (SUVs) were prepared by mixing DOPC, DOPG, DSPE‐PEG(2000) biotin, and Rhodamine B‐PE (or Cy5‐PE) at desired molar percent in chloroform. The solution mixture was first blown with N_2_ for 15 min preceding further drying under vacuum in a desiccator for 3 h. The dried lipid film was then resuspended in PBS buffer by vortexing, resulting in a concentration of about 1 mg/mL. The vesicle solution went through ten cycles of freeze and thaw by being immersed in liquid nitrogen and warm water (∼ 45°C) subsequently. Lastly, the vesicles were extruded through a 100 nm membrane with at least 21 passes, using an Avanti Mini‐extruder. Size of thus synthesized SUVs was measured by dynamic light scattering and is 120 nm ± 20 nm. The SUVs were stored at 4°C and used within 1 week.

When testing for the staining efficiency of membrane probes (COE‐Ben, CellVue Claret or PKH26), the SUVs were incubated with each probe in 1× PBS buffer (pH 7.4) at 37°C for 30 min. The respective final concentrations of SUVs and the membrane probe during the co‐incubation were 0.01 mg/mL and 0.5 µM. Residual probe was cleaned up using an Amicon® Ultra Filter with a molecular weight cut‐off at 100 kDa.

### 2.3 Surface functionalization

Glass coverslips were first cleaned to render the surface hydrophilic and remove residual organic contaminants. Then, a six‐channel ibidi µ‐Slide VI 0.4 was attached onto the cleaned coverslip to form microfluidic imaging chambers. PLL‐g‐PEG‐biotin 50% solution diluted in PBS buffer was introduced into each chamber and was allowed to incubate at 4°C overnight. The chambers were subsequently washed with copious PBS buffer preceding the addition of NeuA proteins at a concentration of 100 µg/mL. NeuA was allowed to incubate on the PLL‐g‐PEG‐biotin surface for 15 min, before washing with PBS buffer.

### 2.4 Isolation and purification of sEVPs

A549 or HEK‐293T (ATCC CCL‐185, or CRL‐3216) were seeded in T175 tissue culture flasks with DMEM + 10% FBS and cells were left to adhere for 24 h in the 5% CO2 chamber at 37°C before changing the media to DMEM with 10% exosome‐depleted FBS (Systems Biosciences, catalog EXO‐FBS‐250A‐1). Cells were grown for 48 h and their health and viability were confirmed prior to media collection for the purpose of minimizing membrane fragments contamination (Théry et al., 2006). The spent cell culture supernatant was carefully collected and filtered using a 0.4 µm PES vacuum filter. sEVPs isolation was then carried out via ultracentrifugation (Optima XPN‐100, Beckman Coulter) at 100,000 × *g* for 1 h at 4°C. The supernatant was carefully removed, and the pellet was resuspended in cold PBS. The sEVPs were pelleted again under the same conditions and then resuspended in 300 µL of PBS. Aliquots of the purified sEVPs were stored at −80°C before downstream analysis. Lyophilized PC‐3 sEVPs derived from cell culture were purchased from Abcam (ab239689). A fresh vial of 100 µg was reconstituted in 1 mL of MiliQ water. The sEVP sample was diluted to 10 µg mL^−1^ using PBS. RBCEVs were generously provided to us by Dr Minh Le at the National University of Singapore. Their isolation and purification were conducted following a published protocol (Usman et al., 2018).

### 2.5 Multidimensional fluorescent labelling of sEVPs

Purified sEVPs were first allowed to incubate with CFSE (5 µM) in 1x PBS buffer (pH 7.4) at 37°C for 30 min. The stained particles were then cleaned up using a size exclusion chromatography (SEC) column (iZon qEVoriginal 70 nm). The concentration and size distributions of these purified particles were characterized using nano‐flow cytometry (Figure S7C). The particle concentration range used for our imaging experiments was 1 × 10^9^/mL to 1 × 10^10^/mL, with the lowest tested concentration being 1 × 10^8^/mL. The size range of purified sEVPs was between 60 nm to 110 nm. This SEC step should remove potential contaminants like membrane fragments from apoptotic cells, because they are larger than sEVPs (Théry et al., 2006). The CFSE‐stained particles were then incubated with PE‐Cy5 labeled CD63 antibodies (400 times diluted from 200 µg/mL) for 30 min at room temperature (24°C ± 1°C) in 1x PBS buffer. The sEVP‐antibody mixture was centrifuged at 9000 RCF for 15 min at 4°C, and 100 µL of the supernatant was introduced into the functionalized imaging chamber for a 1‐h incubation. The chamber was subsequently washed with copious 1x PBS buffer solution to remove any unattached or loosely attached sEVPs, as well as free antibodies. Finally, COE‐Ben (0.5 µM) in 1x PBS buffer was introduced to incubate with sEVPs in the imaging chamber at 37°C for 30 min, followed by thorough buffer wash. The sEVPs arrays formed following this protocol are stable for imaging for at least 24 h at room temperature (Figure S10).

### 2.6 Total internal reflection fluorescence (TIRF) microscopy imaging

TIRF images were acquired at NTU Optical Bio‐Imaging Centre (NOBIC) imaging facilities at SCELSE. TIRF experiments were performed on the Carl Zeiss ELYRA PS.1 / LSM 780 system using the Plan‐Apochromat 63x/1.4 oil‐immersion objective (FWD 0.19 mm, CG 0.17 mm). When imaging in the CFSE channel, the Elyra 488 nm laser line (intensity ∼6 W/cm^2^ at the sample stage) was used with an exposure time of 200 ms and a BP 495–575 + LP 750 emission filter. When imaging in the PE‐Cy5 channel, the Elyra 561 nm laser line (intensity ∼12 W/cm^2^ at the sample stage) was used with an exposure time of 100 ms and a LP 633 emission filter. When imaging in the COE‐Ben channel, the Elyra 405 nm laser line (intensity ∼3 W/cm^2^ at the sample stage) was used with an exposure time of 50 ms and a BP 420–480 + LP 750 emission filter. Fluorescence images were recorded using an EM‐CCD (Andor iXon DU‐897D, 512 × 512 pixels). All acquisitions were obtained using the software ZEN 2012 SP5 FP2, and the subsequent image analysis was conducted with ImageJ. Imaging a 100 µm ×100 µm region of interest (ROI) in three different fluorescence channels takes approximately 3 s. To image the entire 65 mm^2^ chip (∼ 6500 images) would therefore take about 5 h. Analyzing all the data from these 6500 images in a multidimensional manner using our algorithms is expected to take approximately 2 to 3 h.

### 2.7 Colocalization analysis using Fiji plugin—ComDet

The TIRF images were flattened and background corrected preceding analysis in ComDet (version 0.5.5). The flat‐field correction was performed using a Gaussian blur filter with a blur radius of 30 pixels (7.5 µm), and the background correction was performed using a rolling ball algorithm with a radius of 50 pixels (12.5 µm). Images from different channels were first merged to generate a composite image. After convoluting the original image with Gaussian and Mexican hat filter with a size of 2 pixels, the intensity threshold for the convoluted image is set to be between 3*SD and 4*SD. For most samples, we did not observe significant sEVP aggregations, and we segment “larger particles” that appear to be a short string of small particles. In approximately one out of seven samples, large, bright, and spherical sEVP aggregates were observed. Since these aggregates account for only about 2% to 7% of the total particle population in the ROIs, we have excluded them from further analysis. These aggregates are not likely to be a result of ultracentrifugation, as all samples we imaged were subjected to this step as described in the “Isolation and purification of sEVPs” paragraph. Multiple factors may contribute to the random formation of these aggregates, including NeuA aggregation on the surface, contaminants in the solution, degradations in the sEVP sample, among others. An enhanced algorithm for high‐content analysis was developed after colocalization analysis with ComDet. The algorithm can be utilized to analyze particles containing at least two markers and plot two‐dimensional intensity scatter plots between two colocalized markers on individual particles (Figure 5a and 5b). Moreover, it can be used to separately analyse each marker intensity per particle in all subpopulations (Figure 5c and Figure 6). The MATLAB code can be found following this link: https://github.com/Sssimou/high‐content‐analysis‐of‐single‐sEVPs/settings.

### 2.8 Super‐resolution structured illumination microscopy (SR‐SIM)

All SR‐SIM imaging were performed using an alpha Plan‐ Apochromat 100x/1.46 oil DICIII objective lens and pco.edge sCMOS camera fitted onto an Elyra PS.1 microscope (Zeiss). Laser wavelengths of 561, 488, and 405 nm at 20% power with exposure times of 100 ms were used to excite PE‐Cy5, CFSE, and COE‐Ben respectively. Images were acquired using five grid rotations with 51 µm grating period and reconstructed using Zeiss software (ZEN 2012 SP5 FP2, black edition). Images were processed using ImageJ.

### 2.9 Flow cytometry

Flow cytometry experiments were performed on the Beckman Coulter CytoFLEX LX using the VSSC configuration as described in previous literature (Zhou et al., 2023). Briefly, the blue SSC (488 nm) was modified to VSSC (405 nm) by moving the 405/10 VSSC filter to the V450 channel in the wavelength‐division multiplexing. Latex beads (100 and 300 nm) were used for calibration. The gain setting for the V405 channel (VSSC) was set to 450. All other channel gains were set to 3000. Detection was triggered on the VSSC > 4000. The fluorescence of COE‐Ben was measured using a spare 450 nm bandpass filter in place of the UV675 channel. Before the acquisition of samples, the instrument was washed for 15 min with Contrad 70 detergent (Beckman Coulter) and for another 30 min using deionized (DI) water. Once events for DI water were low (< 1000 µL^−1^), the instrument was deemed ready for data collection. Samples were boosted by running for 1 min on the high flow rate (60 µL min^−1^) and then changed to slow (10 µL min^−1^). Data collection was terminated by controlling the sample volume (10.0 µL). Data were analysed using CytExpert software. As shown in the resulting figures, positive gates were set to exclude events from the small EV–only sample.

### 2.10 Nano‐flow cytometry

A NanoFCM NanoAnalyzer U30 instrument, equipped with 488 nm and 638 nm lasers, was employed for size and concentration measurements via side scatter detection (NanoFCM Inc., Nottingham, United Kingdom). The instrument underwent calibration per the manufacturer's protocols, employing 250 nm fluorescent quality control beads of known concentration and S16M‐Exo sizing beads comprising four distinct‐sized silica nanospheres (NanoFCM Inc.) with diameters of 68, 91, 113, and 155 nm. A standard curve was generated based on the side scattering intensity of the sizing beads using the NanoAnalyzer software, and this standard curve was employed for size determinations of our samples. Before sample analysis, the instrument underwent thorough washing, utilizing ultrapure water and cleaning fluid provided by the manufacturer. All samples were diluted 1 x PBS buffer before being loaded onto the instrument and the dilution was recorded in the software for concentration calculations. The samples were measured under small threshold settings (68‐155 S16M‐Exo). Sizing and concentrations were determined using the NanoAnalyzer software.

### 2.11 Negative‐stain transmission electron microscopy (TEM)

The sEVP sample was adsorbed to carbon‐coated 200 mesh copper grids (Electron Microscopy Sciences) and blotted with filter paper and negatively stained with 4 µL of 2% (w/v) uranyl acetate solution. Images were collected using a Tecnai T12 (Thermofisher Scientific) transmission electron microscope operated at 120 kV using a 4k×4k Eagle (Thermofisher Scientific) CCD camera at a nominal magnification of 30000× and a de‐focus value of ‐5.054 µm. The calibrated pixel size was 368 pm at the specimen level.

## 3 RESULTS

Utilizing a universal optical reporter that unbiasedly labels the majority, if not all, of sEVPs is an ideal strategy, and one commonly employed approach is to fluorescently stain the lipid bilayers. The assumption is that the non‐vesicular NPs constitute an inconsequential fraction of the total sEVP population, hence the lipid bilayer, being a hallmark component of vesicles, serves as a normalization basis for comparison across different samples. Presently, widely used membrane probes are primarily lipophilic carbocyanine–based compounds, with notable examples including the Di and PKH families (Zhou et al., 2023). However, it has been recently observed that these dyes tend to aggregate into particles of sizes akin to sEVPs in aqueous environments, potentially generating false‐positive signals (Pužar Dominkuš et al., 2018). One important advancement in the field is the development of a family of water‐soluble membrane dyes known as COEs, and they successfully labelled lipid bilayers of vesicles, exosomes and cellular membranes (Zhou et al., 2023). In order to determine a robust membrane probe for staining sEVPs in our high‐content quantitative measurements, we set out to characterize and compare the labelling efficiency of PKH26, CellVue Claret, and COE‐Ben for lipid bilayers using synthesized small unilamellar vesicles (SUVs). These particles are used as an initial control because they have well‐defined membrane structures.

**FIGURE 2:**
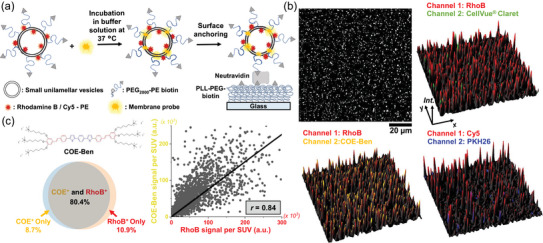
Identification and quantification of a robust fluorescent marker for lipid membranes. (a) Schematic demonstration of staining fluorescent SUVs (containing either RhoB‐PE or Cy5‐PE) with water‐soluble membrane probes (CellVue Claret, PKH26, or COE‐Ben) before surface anchoring to a PLL‐PEG‐biotin and neutravidin functionalized substrate for TIRF imaging. (b) Representative TIRF image (grayscale) and 3D intensity plots (two‐channel merged) of SUVs stained with both water‐soluble membrane probes and fluorescent lipid‐dye conjugates. (c) Colocalization analysis of the RhoB intensities and the COE‐Ben intensities per SUV, including quantification of subpopulations and intensity scatter plots of the two fluorescent markers. The composition of SUVs is: 0.025 mol% RhoB‐PE, 0.5 mol% biotin‐PEG2000‐DSPE, 10 mol% DOPG, and 89.475 mol% DOPC, or 0.5 mol% Cy5‐PE, 0.5 mol% biotin‐PEG2000‐DSPE, 10 mol% DOPG, and 89 mol% DOPC. Three individual imaging experiments were performed with three different batches of SUV samples. Seven 100 µm × 100 µm regions were randomly selected, and all 6015 particles in these regions were sampled and analysed.

During the process of SUV synthesis, lipid‐dye conjugates (Rhodamine B‐PE, referred to as RhoB‐PE hereafter, or Cy5‐PE) and biotin‐PEG conjugated lipids were introduced alongside other major lipid components in the organic phase (detailed description of SUV synthesis is provided in the Methods section). The signal from lipid‐dye conjugates serves as the positive control for SUVs, whereas the presence of biotin‐PEG conjugated lipids facilitates the anchoring of SUVs onto a surface for TIRF imaging (Figure 2a, Figure S1). To prepare the SUV‐capturing surface, we first functionalize a glass substrate with poly(L‐lysine (PLL)‐PEG‐biotin, followed by the addition of NeutrAvidin (NeuA). SUVs were incubated separately with the three membrane probes in the aqueous buffer solution prior to surface attachment (detailed staining procedure is provided in the Methods section). TIRF microscopy on the subsequently formed single SUV arrays allows us to measure thousands of SUVs in parallel, and we image the intensity signal per SUV in both the lipid‐dye conjugate and membrane probe channels (Figure 2b, Figure S2A). Using a Fiji Plugin—ComDet (https://github.com/UU‐cellbiology/ComDet), we detect each SUV that appears as a diffraction‐limited bright spot and colocalize the intensity spots of the two fluorescent labels. We find that the intensities of both CellVue Claret and PKH26 poorly colocalize with the RhoB‐PE (or Cy5‐PE) intensities. In fact, less than 3% of the total particle population (*N* = 2992 particles) exhibit co‐staining (Figure S2B). This observation suggests that only a small proportion of lipid vesicles is effectively labelled using CellVue Claret or PKH26, aligning well with a recent publication (Melling et al., 2022).

In stark contrast, the intensities of COE‐Ben demonstrate exceptional colocalization with those of RhoB‐PE (Figure 2b). 80.4% of the total particle population (*N* = 6015 particles) contains both COE‐Ben and RhoB‐PE. 8.7% have only COE‐Ben, whereas 10.9% have only RhoB‐PE (Figure 2c, see Figure S2 for further discussion). At the concentrations used here, COE‐Ben itself does not form any observable aggregates (Figure S3), and both RhoB and COE‐Ben should reach their maximum staining ratios (defined as stained particle number / total particle number, see Figure S4 for detailed discussion). Hence, it is reasonable to conclude that COE‐Ben stains ∼ 90% (80.4% + 8.7%) of the total detectable SUV population. Furthermore, within the population that contains both labels, we map the integrated intensities of COE‐Ben and RhoB per SUV in a two‐dimensional scatter plot and find a relatively strong positive correlation (Figure 2c). The number of RhoB‐PE molecules incorporated in the membrane is proportional to the SUV surface area and thereby related to the particle size (Mathiasen et al., 2014). Control experiments in Figure S5A demonstrate that the average COE‐Ben signal per SUV grows linearly with the average size of the vesicle measured by dynamic light scattering (DLS). Moreover, the distribution of the square root of COE‐Ben integrated intensity per SUV is consistent with the SUV size distribution (Figure S5B and S5C) (Jiang et al., 2021). Together with the result of positive correlation between RhoB‐PE and COE‐Ben signals, we conclude that the fluorescence intensity of a COE‐Ben‐stained SUV is proportional to the surface area of the lipid membrane. Therefore, we establish that COE‐Ben, as a water‐soluble fluorescent probe, stains lipid membranes robustly and quantitatively comparable to standard membrane markers that require preparation in the organic phase. We decide to employ COE‐Ben for labelling lipid membranes in sEVPs.

**FIGURE 3:**
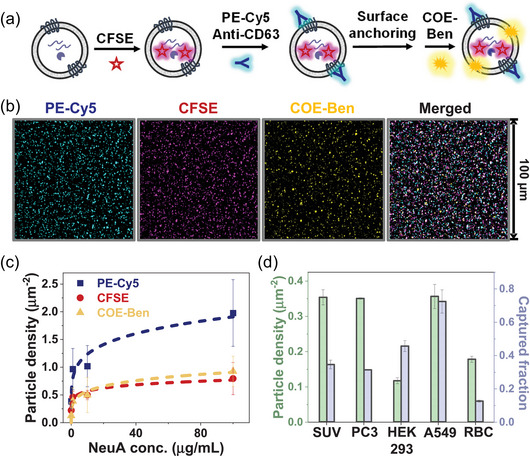
Demonstration of the entire workflow for fluorescent labelling and surface anchoring of sEVPs that enables high‐content TIRF imaging at the single‐particle level. (a) Schematic illustration of the staining order to label sEVPs with three different fluorescent markers—CFSE, PE‐Cy5 labelled CD63 antibodies, and COE‐Ben. (b) Representative TIRF images of PC3 sEVPs in the three fluorescent channels, shown as both individuals and as merged. (c) Density of surface‐anchored sEVPs, respectively detected in the three fluorescence channels, are plotted as a function of NeuA concentration. Two individual imaging experiments were performed with two different batches of PC3 sEVP samples for each NeuA concentration. At each NeuA concentration, eight 100 µm x 100 µm regions were randomly selected, and all particles in these regions were sampled to calculate the average particle density. Error bars represent standard error among selected regions. (d) Surface densities and captured fractions of biotin‐containing SUVs and sEVPs derived from PC3, HEK293, A549, and RBC. sEVPs in all three fluorescence channels are included. Two individual imaging experiments were performed with one batch of SUV, PC3 sEVP, HEK sEVP, A549 sEVP, and RBC sEVP. For each sample, six 100 µm × 100 µm regions were randomly selected, and all particles in these regions were sampled to calculate the average particle density and the corresponding captured fraction. Error bars represent standard error among selected regions.

To visualize the structural features of sEVPs, which are necessary to understand their biological functions, we employ a multiplexed labelling approach that targets the outer surface, membrane plane, and luminal space of the particles. Specifically, the outer surface was labelled using a PE‐Cy5 labelled CD63 antibody. CD63, a transmembrane protein belonging to the tetraspanin family, was selected due to its notably high abundance within sEVPs (Jeppesen et al., 2019). The integrated PE‐Cy5 intensity per particle should directly correlate with CD63 copy number per particle. Carboxyfluorescein succinimidyl ester (CFSE) serves as the luminal marker: It can penetrate through the outer layer of sEVPs and fluoresce upon cleavage by luminal esterases. It will then covalently bind to free amine groups on proteins (Lyons, 2000). CFSE has been one of the most widely used internal markers for sEVPs, and its robustness is well‐documented using a variety of techniques (Fortunato et al., 2021; Lau et al., 2024; Woud et al., 2022). Therefore, we consider CFSE‐labelled proteins as representative cargoes of sEVPs, and the integrated CFSE intensity per particle should directly correlate with both esterase and CFSE contents in the particle. Moreover, COE‐Ben is used to label the plane of lipid membrane and distinguish vesicular and non‐vesicular particles within the sEVP sample (given that the membrane composition of sEVs can differ greatly from synthesized SUVs, it is anticipated that the staining efficiency of COE‐Ben on sEVs will vary from what was quantified in Figure 2). To evaluate the staining performance of CFSE and COE‐Ben, in Figure S6, we provide their positive event rates measured by flow cytometry. Collectively, we aim to achieve comprehensive fluorescence labelling of the total sEVP population. Figure 3a provides a brief overview of the multidimensional staining procedure, and detailed description of this procedure is provided in the Methods section. Briefly, the isolation and purification of sEVPs from cell culture follows established protocols (Zhou et al., 2023) (see details in the Methods section, and the quality of the purified sEVPs was verified using multiple techniques (Welsh et al., 2024), including electron microscopy, EV surface markers, and nano‐flow cytometry (Figure S7)). Purified sEVPs were allowed to incubate with CFSE in PBS buffer at 37°C for 30 min. After cleaning up the residual dye using a size exclusion column, CFSE‐treated particles are incubated with PE‐Cy5 labelled CD63 antibodies prior to surface anchoring. Finally, COE‐Ben is introduced to the anchored particles within the imaging chamber, followed by thorough buffer washing. It is important to note that this specific staining order is critical for obtaining high‐quality fluorescence images of individual sEVPs (see detailed discussion in Figure S8). An additional advantage of this multidimensional labelling scheme is the minimized FRET effect due to the spatial segregation of the multiple fluorescent markers (Figure S9).

Next, we establish a capturing platform with little bias for imaging the fluorescently labelled sEVPs using TIRF. Interestingly, we find that sEVPs can stably attach to the PEG‐NeuA coated glass surface without the need for additional capture antibodies (Figure 3b and Figure S10). The density of attached sEVPs can be tuned by modulating the concentration of NeuA; without NeuA, sEVPs exhibited minimal adsorption to the polymer‐coated substrate (Figure 3c and Figure S11B). Moreover, the density of particle arrays is mostly uniform across a 3.8 mm × 17 mm imaging chamber (Figure S12). Using super‐resolution structured illumination microscopy (SR‐SIM), we confirm that the majority of surface‐captured sEVPs are single particles (Figure S13). Moreover, we compare the intensity histograms of COE‐Ben signal per particle when two different NeuA concentrations are used (10 and 100 µg/mL) (Figure S14), and they demonstrate only negligible difference. This result further supports that sEVPs are prone to anchor at the surface binding sites as individual particles instead of forming aggregates. Notably, when we coated the PEG‐NeuA surface with a layer of biotinylated CD63 antibodies prior to sEVP attachment, we observed a decreased sEVP capture rate, and these antibodies seemed to induce some extent of sEVP aggregation (Figure S15). It is important to note that protein‐mediated sEVP attachment is not specific to NeuA—bovine serum albumin (BSA) can also effectively capture sEVPs (Figure S11). These nonspecific interactions between sEVPs and the anchoring proteins are, at least in part, due to electrostatic attractions (Figure S11B) and are reminiscent of mechanisms involved in the formation of a protein corona on the surface of EVs (Tóth et al., 2021). Therefore, while our capturing platform is likely less biased than tetraspanin antibody‐functionalized surfaces (as it can capture particles without requiring tetraspanins), it may not be completely bias‐free. All subsequent imaging experiments were performed on the NeuA surface, as the nearly neutral charge of NeuA at the experimental pH is expected to minimize unwanted non‐specific interactions. Using a NeuA concentration of 100 µg/mL, where sEVP densities in the three marker channels reach a plateau, we quantify the surface density and calculate the captured fraction of all detectable sEVPs from four cell types in Figure 3d. These include PC3 ‐ human prostate cancer cell, HEK293 ‐ immortalized human embryonic kidney cell, A549 ‐ adenocarcinomic human alveolar basal epithelial cell, and RBC—red blood cell (while representative fluorescence images of PC3 sEVPs are shown in Figure 3b, results with the other three types of sEVPs are provided in Figure S16). The captured fraction represents the portion of sEVPs from the bulk solution (measured by flow cytometry, and the calculation process for captured fractions is demonstrated with Figure S6) that are immobilized on the functionalized surface, and we compare the results from sEVPs with that of SUVs containing biotin‐PEG conjugated lipids. The SUVs demonstrate a saturated surface density of 0.37 particles/µm^2^, indicating the maximum level of attachment on the NeuA‐decorated surface. The captured fraction for these SUVs is calculated to be 0.35. In the case of sEVP samples, their captured fractions on the NeuA surface ranges from 0.12 to 0.7, comparable to that of biotin‐SUVs. Such capture efficiency for sEVPs is notably higher than some reported for antibody‐coated surfaces (Daaboul et al., 2016; Lee et al., 2018; Saftics et al., 2023) and is comparable to an optimized commercial chip (ExoView chip functionalized with CD81 antibodies) (Mizenko et al., 2021) and some functionalized nanostructured surfaces (Yao et al., 2023; Yasui et al., 2021). It is noteworthy that the captured fractions we calculate here is relative and dependent on the sEVP bulk concentration—once the surface binding sites for sEVPs are saturated, increasing sEVP bulk concentration leads to decreased capture efficiency (Figure S17). Moreover, with a surface density ranging from 0.12 particles/µm^2^ to 0.36 particles/µm^2^ on a 65 mm^2^ imaging chip, we could image and analyze approximately 1.5 × 10^7^ particles in a single experiment. Thus, we establish a workflow to robustly label sEVPs in a multidimensional manner and to conduct high‐throughput imaging of sEVP arrays by intact immobilization with little bias onto a soft material‐functionalized surface.

**FIGURE 4:**
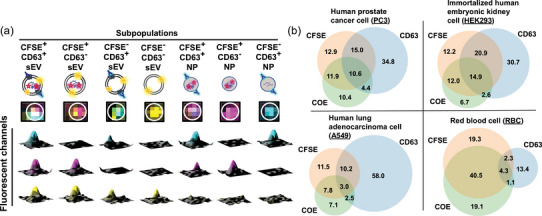
Identification and characterization of distinct subpopulations within sEVP samples. (a) Demonstration of the seven distinct subpopulations when sEVPs are labelled with three fluorescence markers in a multidimensional manner. The 3D intensity surface maps in the three fluorescent marker channels plotted under the same column belong to one representative particle of a specific subpopulation. (b) Venn diagrams of subpopulation fractions in four different sEVP samples. At least three individual imaging experiments were performed with three batches of PC3, HEK, A549, and RBC sEVP samples. For each sample, ten to fifteen 100 µm × 100 µm regions were randomly selected, and all particles (31380 particles for PC3, 21200 particles for HEK, 23411 particles for A549, and 20183 particles for RBC) in these regions were sampled and analysed.

As visually demonstrated by the merged three‐channel TIRF images in Figure 3b and Figure S16, the four types of sEVP samples exhibit vast heterogeneity regardless of their cellular origins. We first seek to quantitatively assess the populational heterogeneity within sEVPs. For each sample, we image $2\times 10^{4}$ to $3\times 10^{4}$ particles and conduct colocalization analysis of intensity spots in the PE‐Cy5, CFSE, and COE‐Ben channels using ComDet. Remarkably, across all four sEVP types, the particles can be categorized into seven distinct subpopulations (Figure 4a). These subpopulations can be further grouped into two subsets based on whether they can be stained by COE‐Ben: the COE‐Ben‐positive particles are categorized as vesicular sEVs, while those COE‐Ben‐negative ones are designated as non‐vesicular NPs. Negative‐stain transmission electron microscopy (TEM) was employed to confirm the presence of both vesicular and non‐vesicular particles in all four types of sEVP samples (Figure S7). Among sEVs, we designate particles that contain all three markers as “CFSE**^+^** CD63**^+^** sEVs.” Those with luminal markers but without CD63 markers are termed as “CFSE**^+^** CD63**^−^** sEVs,” and those with CD63 but without CFSE are considered “CFSE**^−^** CD63**^+^** sEVs.” We also detect some “CFSE**^−^** CD63**^−^** sEVs that lack both CFSE and CD63 signals.” Within the NP category, those containing both luminal and CD63 markers are referred to as “CFSE**^+^** CD63**^+^** NPs.” Additionally, particles containing only CFSE or only CD63 are recognized as “CFSE**^+^** CD63**^−^** NPs” and “CFSE**^−^** CD63**^+^** NPs,” respectively. Control experiments demonstrate that the number density of COE‐Ben‐positive particles reaches a plateau level at the concentration of COE used here (Figure S18), suggesting that the majority of vesicular particles should have been labelled with COE‐Ben. Next, we characterize the non‐vesicular NPs. We find that these NPs are unlikely to be contaminants like lipoproteins (Figure S19). Moreover, the CFSE^+^ NPs should contain active esterases. As for the CFSE**^−^** CD63^+^ NPs, they are unlikely to consist of antibody aggregates present in or induced by the cell culture media (Figure S20A and S20B) and are most likely not random protein aggregates based on results from Triton X‐100 treatment (Figure S20C and S20D, detailed discussion is provided in the figure legend). Additionally, we observe that proteinase K treatment of the sEVPs does not exclusively degrade NPs; it causes population decreases across all subtypes, except for the CFSE**^−^** CD63**^−^** sEVs (Figure S21). Collectively, we propose that the vast majority of NPs imaged here are unlikely to be artificial or cell‐derived protein aggregates.

For each type of sEVPs, the fractions of the seven subpopulations are plotted in a Venn Diagram (Figure 4b, error bars provided in Table S1). Notably, distinct subpopulation distributions are observed among samples secreted by different cell types, implying the presence of unique cellular fingerprints. Also, we find that the fraction of CFSE**^+^** CD63^+^ sEVs in the total particle population is surprisingly low, from 3.0% in the A549 sEVPs to 14.9% in the HEK293 sEVPs. Moreover, we calculate that the total fraction of sEVs ranges from 20.4% in the A549 sEVPs to 65% in the RBC sEVPs, underscoring the importance of acknowledging the non‐vesicular NPs in sEVPs prepared using conventional protocols. Additionally, we notice some interesting statistics in both the sEV and NP subsets. Within the sEVs, CFSE**^−^** CD63**^−^** sEVs can account for up to 30% of the population, indicating that loading efficiency should be an important parameter to consider in engineering sEVPs for drug delivery. Moreover, within NPs, while the fractions of CFSE**^+^** CD63**^−^** NPs remain comparable across the four cell types (ranging from 21.7% to 33.1%), the proportion of CFSE**^−^** CD63**^+^** NPs varies substantially, from 15.7% in the RBC sEVPs to 68.2% in the A549 sEVPs. This result may suggest divergent biogenesis pathways for these two NP subpopulations.

**FIGURE 5:**
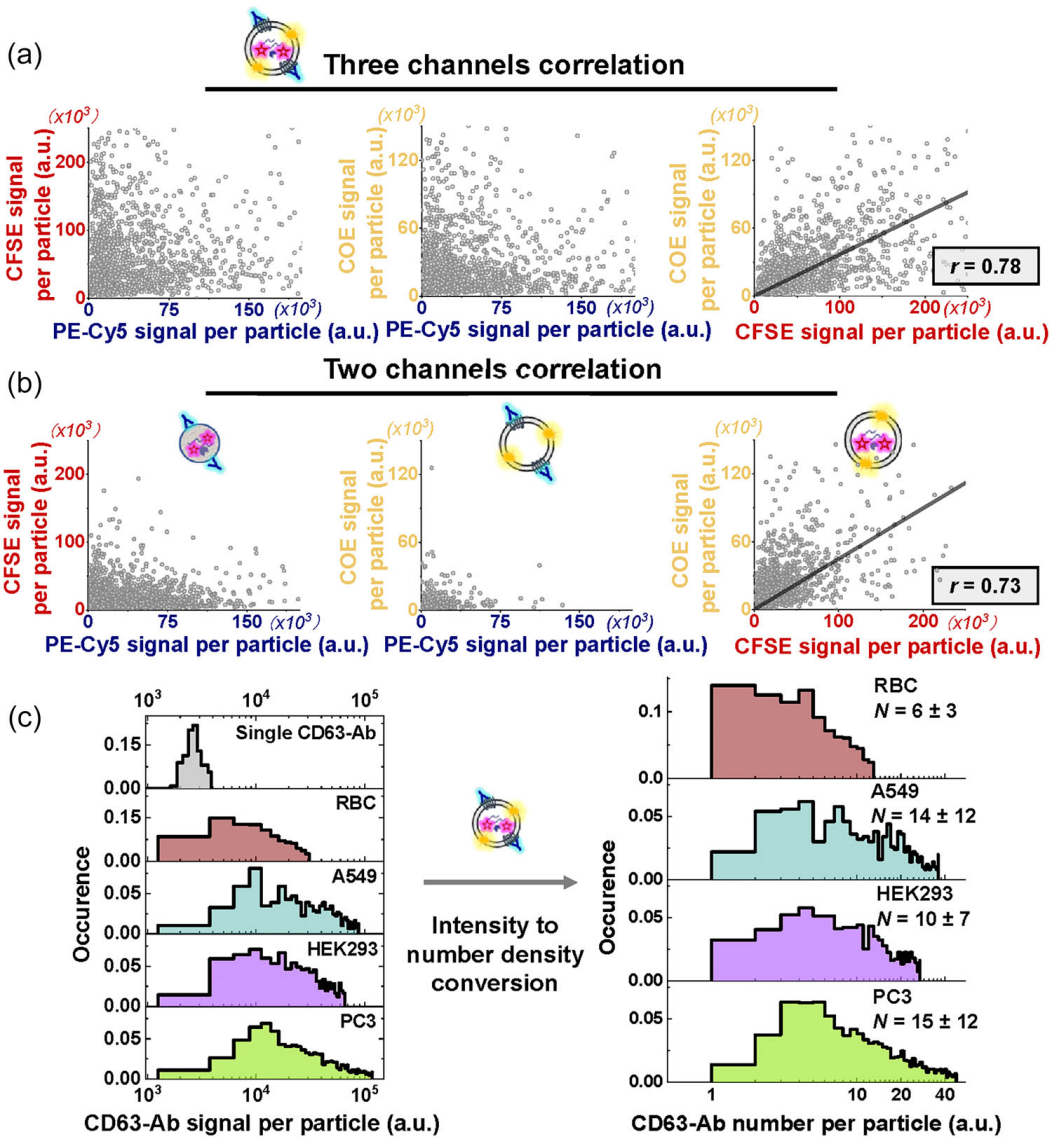
Quantitative analysis of intensity statistics in the three fluorescent labels on individual sEVPs from different subpopulaitons. (a) Intensity scatter plots between each two of the three colocalized fluorescent markers in CFSE**^+^** CD63^+^ sEVs (include 1432 particles from two representative trials). (b) Intensity scatter plots between two colocalized fluorescent markers in CFSE**^+^** CD63**^+^** NPs, CFSE**^−^** CD63^+^ sEVs, and CFSE**^+^** CD63**^−^** sEVs, respectively (include 2314, 298, and 1150 particles from two representative trials, respectively). (c) Intensity histograms of single PE‐Cy5 labelled CD63 antibodies and PE‐Cy5 in individual CFSE**^+^** CD63^+^ sEVs are used to calculate histograms of CD63 count per CFSE**^+^** CD63^+^ sEVs. Particle intensity profiles from all trials are included here.

Next, we aim to quantitatively characterize the compositional heterogeneity within each subpopulation of sEVPs. Utilizing ComDet as a foundation, we develop an enhanced algorithm to perform deep profiling of the content within each sEVP (Github link is provided in the Methods section). This is achieved by analysing intensity statistics of spots across the three fluorescent marker channels. In Figure 5, we characterize the integrated intensity of each marker on individual PC3 sEVPs and generate two‐dimensional intensity scatter plots for subpopulations containing a minimum of two markers (Results from the other three sEVP types are provided in Figure S22, and they exhibit qualitatively similar characteristics). In Figure 5a, we analyze CFSE**^+^** CD63^+^ sEVs that contain all three colocalized markers and generate three intensity scatter plots (The impact from batch‐to‐batch variation of staining efficiency is negligible, as illustrated in Figure S23). The CFSE signal per particle demonstrates a relatively strong positive correlation with the COE‐Ben signal, while the CD63 signal shows a poor positive correlation with either CFSE or COE‐Ben (Figure 5a). Given our previous demonstration that the COE‐Ben signal per vesicular particle is proportional to the surface area of the lipid membrane (Figure S5), it can be inferred that the luminal protein content in CFSE**^+^** CD63^+^ sEVs is likely proportional to their sizes as well. Utilizing nano‐flow cytometry, we confirm that the CFSE signal per particle indeed increases with growing sEVP size (Figure S24). Moreover, it appears that sorting of CD63 in CFSE**^+^** CD63^+^ sEVs follows a different rule. This could be partially explained by the recently observed curvature‐sensitive characteristic of tetraspanins (Dharan et al., 2022; Walsh et al., 2018). In simpler terms, the preference for highly curved membranes might lead to a higher probability for smaller sEVs to incorporate more CD63 per particle than larger sEVs. Furthermore, we profile an intensity scatter plot for each sEVP subpopulation containing two out of the three fluorescent markers (Figure 5b). Specifically, in CFSE**^+^** CD63**^+^** NPs (CFSE**^+^** and CD63**^+^** only, left panel), the CFSE signal and CD63 signal per particle display a weak positive correlation. In CFSE**^−^** CD63**^+^** sEVs (COE**^+^** and CD63**^+^** only, middle panel), the COE‐Ben signal and CD63 signal per particle also demonstrate a weak positive correlation. Additionally, in CFSE**^+^** CD63**^−^** sEVs (CFSE**^+^** and COE**^+^** only, right panel), the CFSE signal and COE‐Ben signal per particle exhibit a relatively strong positive correlation, albeit with a correlation coefficient lower than that of CFSE**^+^** CD63^+^ sEVs.

Despite being considered one of the most reliable markers for sEVPs, the exact number of CD63 molecules per sEVP shows a large discrepancy in literature (Corso et al., 2019; Puthukodan et al., 2023). We argue that it is important to accurately quantify this number, because CD63 was found to contribute to exosome biogenesis (Sung et al., 2020) and contradictory results were reported regarding the correlation between CD63 level and the cancerous state of sEVPs (Song et al., 2020; Yoshioka et al., 2013). We first seek to characterize the CD63 count per CFSE**^+^** CD63^+^ sEV in the sEVPs derived from the four cell types. Histograms of integrated PE‐Cy5 intensity per CFSE**^+^** CD63^+^ sEV are displayed in Figure 5c, together with an intensity histogram of single PE‐Cy5 labelled CD63 antibodies (uppermost panel, solid grey). The histogram for single antibodies is constructed through imaging surface‐immobilized antibodies in the absence of sEVPs, with their monomeric state confirmed via single‐step photobleaching (Figure S25A, see detailed discussion on the method in the figure legend) (Huang et al., 2016). We fit a Gaussian function to the single‐antibody intensity histogram and utilize the peak position as the average “single‐CD63 signal.” It is worth noting that the amount of non‐specifically adsorbed CD63 antibodies is negligible compared to the amount of captured sEVPs (Figure S25C). Therefore, by dividing the histograms of integrated PE‐Cy5 intensity per CFSE**^+^** CD63^+^ sEV by the average value of single‐CD63 signal, we can convert the intensity histograms to histograms of CD63 count per particle. We also applied a previously reported intensity deconvolution method to calculate the average number and number distribution of CD63 per particle (Figure S26) (Mutch et al., 2011). The results are comparable with Figure 5c. Clearly, the CD63 number per CFSE**^+^** CD63^+^ sEV is substantially heterogeneous, even among those originating from the same cell type. Noting that batch‐to‐batch variations should have only be a minor impact on such heterogeneity (Figure S23). In addition, the average count and heterogeneity of CD63 per CFSE**^+^** CD63^+^ sEV derived from the two cancer cell types (PC3 and A549) is notably higher than those in CFSE**^+^** CD63^+^ sEV derived from the two noncancerous cell types (RBC and HEK293).

**FIGURE 6:**
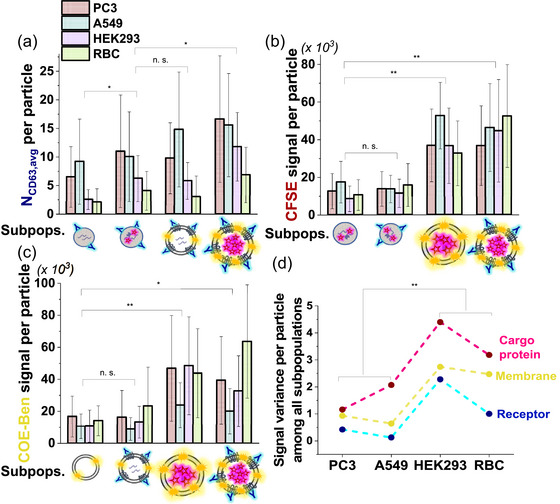
Average signal of the three fluorescent labels per sEVPs in various particle subpopulations derived from four cell types (a) Average CD63 number per particle in four CD63‐positive sEVP subgroups. (b) Average integrated CFSE fluorescence intensity per particle in four CFSE‐positive sEVP subgroups. (c) Average integrated COE‐Ben fluorescence intensity per particle in four COE‐Ben‐positive sEVP subgroups. (d) Signal variance of the normalized intensity of each fluorescent label among all sEVPs subpopulations from four cell types. *p < 0.05, **p < 0.01. At least three individual imaging experiments were performed with three batches of PC3, HEK, A549, and RBC sEVP samples. For each sample, ten to fifteen 100 µm × 100 µm regions were randomly selected, and all particles (31380 particles for PC3, 21200 particles for HEK, 23411 particles for A549, and 20183 particles for RBC) in these regions were sampled and analysed. Error bars represent standard error among different trials.

We next characterize the CD63 molecular copy number per particle within the four sEVP subpopulations containing PE‐Cy5 signals (corresponding intensity histograms are provided in Figure S27). Their respective average CD63 counts are depicted in Figure 6a, and the numbers are consistently higher (*p* < 0.001) in the two cancerous sEVPs (maroon: PC3, blue: A549) than those in the two noncancerous sEVPs (purple: HEK293, green: RBC), regardless of the subpopulation type. This quantitative difference becomes most pronounced in the CFSE**^−^** CD63**^+^** NP population. Moreover, similarly observed across the four different cell types, the CD63 count per particle in the CFSE**^−^** CD63**^+^** NPs is significantly lower than that in CFSE**^+^** CD63**^+^** sEVs. Meanwhile, the CFSE**^+^** CD63**^+^** NPs and CFSE**^−^** CD63**^+^** sEVs have comparable CD63 count per particle and both are slightly lower than that in CFSE**^+^** CD63**^+^** sEVs. Furthermore, we profile the integrated intensity of CFSE or COE‐Ben per particle within the sEVP subpopulations containing CFSE or COE‐Ben, respectively (Figure 6b and 6c, with corresponding histograms provided in Figure S27). Among the CFSE‐positive particles (Figure 6b), the two sEV populations show substantially stronger CFSE signal per particle compared to the two NP populations. Within the COE‐Ben‐positive particles (Figure 6c), the two CFSE**^+^** sEV populations exhibit an overall stronger COE‐Ben signal per particle than the other two CFSE**^−^** sEV populations. Given that we have demonstrated that both CFSE and COE‐Ben signals should be proportional to particle sizes (Figure S5 and S24), it is reasonable to conclude that sEVs containing luminal proteins tend to have larger sizes than NPs, aligning well with recent cryo‐EM findings (Zhang et al., 2018; Zhang et al., 2021). Moreover, sEVs containing luminal proteins consistently appear larger than their luminal protein‐free counterparts. Intriguingly, these patterns remain consistent across the four different cell types. Additionally, we can quantitatively describe the signal heterogeneity of each marker among different subpopulations by calculating the variance of their normalized intensity across varied subpopulations (Figure 6d). A larger variance indicates that signals among the subpopulations are more dispersed, hence greater heterogeneity. This heterogeneity can be potentially important for understanding biogenesis and disease‐associated properties of sEVPs (Jeppesen et al., 2019; Von Lersner et al., 2024). Notably, it appears that the variances of all three markers are consistently smaller in the two cancerous sEVPs compared to the two noncancerous sEVPs that we tested.

## 4 DISCUSSION

In this study, we employ direct TIRF imaging to investigate sEVP arrays, mapping their inherent heterogeneity at the single‐particle level. Through multiplexed and spatially segregated fluorescent labelling, surface anchoring with little bias, and a robust colocalization and deep profiling workflow, we distinguish seven distinct subpopulations of particles in sEVP samples derived from four cell types. Moreover, the quantitative distribution of these subpopulations proves to be cell‐type dependent. Surprisingly, our findings demonstrate that CFSE**^+^** CD63**^+^** sEV, such as exosome, (Jeppesen et al., 2019) is not the dominant constituent within sEVP samples. In fact, vesicular and non‐vesicular particles appear to exhibit comparable populations. Based on results in Figure 6 and Figure S7, we hypothesize that some of the non‐vesicular NPs we observed could be the recently identified exomeres and supermeres (Zhang et al., 2018, 2021). Further exploration is needed to identify the origin of these NPs. This observation urges reevaluation of the attributed functional consequences of “exosomes”—it is crucial to identify the key subpopulation(s) driving in vivo pathological and therapeutic effects of sEVPs. We believe that membrane fragments are unlikely to contribute significantly to the observed vesicular population, because: (1) we have minimized potential contamination from membrane fragments during the sEVP isolation and purification process, following established protocols (see details in the Methods section) (Théry et al., 2006); and (2) protein‐free membrane fragments are not expected to attach well to the NeuA surface (Figure S1). In addition, we conduct a comprehensive and quantitative characterization of the multi‐dimensional content of individual sEVPs within each of the seven subpopulations. First, we unveil common features in population‐dependent cargo sorting. For example, CFSE**^+^** CD63**^+^** sEVs display a pronounced cargo protein and tetraspanin content, accompanied by relatively larger particle sizes compared to the remaining six particle types. Next, we demonstrate the potential utility of distinct sEVP features in discriminating between sEVPs derived from two types of cancer cells and two types of noncancerous cells. For example, the cancerous sEVPs exhibit a higher average CD63 count per particle, coupled with greater heterogeneity. Additionally, the signal variance of identical fluorescent markers among different subpopulations consistently appears higher in noncancerous sEVPs than in cancerous counterparts. This indicates that quantitative content disparities across diverse sEVP subpopulations may be compromised in cancer cells. Indeed, recent recognition of cancer cells' manipulation of exosome biogenesis machinery reinforces this observation (Han et al., 2022). Collectively, the quantitative analysis presented in our study offers valuable insights into the biophysics and biogenesis of sEVPs, as well as implications for sEVP‐based theranostics.

We expect the imaging platform and data analysis workflow established here to serve as a powerful and fascile tool driving advancements in the field of sEVP research. For example, a logical extension of our current labelling scheme is to utilize multiple fluorescent labels targeting disease‐associated biomarkers that are spatially segregated on sEVPs and to conduct deep profiling of these biomarkers. Moreover, a comprehensive and quantitative database regarding material attributes of individual pathological sEVPs from biological samples can be constructed, allowing the development of a high‐throughput, imaging‐based sEVP diagnostic assay. In addition, functional consequences of each sEVP subpopulation can potentially be decoded. For example, cells can be introduced to interface with the functionalized surface, enabling direct, controllable, and single‐particle level explorations of processes such as sEVP release from donor cells and their subsequent uptake by recipient cells. Furthermore, a high‐throughput, imaging‐based screening assay can be developed for improving the specificity of engineered sEVPs, as both therapeutic agents and drug‐delivery vessels.

## CONFLICT OF INTEREST STATEMENT

G.C.B. and S.J.C.‐V. are inventors on a patent application related to this work filed by the National University of Singapore (no. PCT/SG2022/050582, filed 16 August 2022). S.S., G.C.B., and J.T.G are inventors on a provisional patent application related to this work filed by Nanyang Technological University (10202400399P, filed 14 February 2024). The authors declare that they have no other competing interests.

## Supporting information



Supporting Information

## Data Availability

All data needed to evaluate the conclusions in the paper are present in the paper and/or the Supplementary Materials.
