# Supplementary material for: Direct imaging with multidimensional labelling and high‐content analysis allows quantitative categorization and characterizations of individual small extracellular vesicles and nanoparticles (sEVPs)
Source: J Extracell Vesicles. 2024 Dec 12;13(12):e12520. doi: 10.1002/jev2.12520 (PMC11635478; doi:10.1002/jev2.12520)
Supplement: Supplementary file 1 — Supporting Information [file JEV2-13-e12520-s001.pdf]

Supplemental Materials for "Direct Imaging with Multidimensional Labeling and High-Content Analysis Allows Quantitative Categorization and Characterizations of Individual Small Extracellular Vesicles and Nanoparticles (sEVPs)"

Simou Sun,[1] Sarah J. Cox-Vázquez,[2,3] Nam-Joon Cho,[4] Guillermo C. Bazan,[1,2,3] Jay T. Groves[1,5#]

[1]Institute for Digital Molecular Analytics and Science, Nanyang Technological University, Singapore 636921, Singapore

[2]Department of Chemistry, National University of Singapore, Singapore 117543, Singapore

[3]Institute for Functional Intelligent Materials, National University of Singapore, Singapore 117544, Singapore

[4]School of Materials Science and Engineering, Nanyang Technological University, Singapore 639798, Singapore

[5]Department of Chemistry, University of California, Berkeley, Berkeley, CA 94720, USA

[#]Correspondence: jtgroves@lbl.gov

Supporting Figures

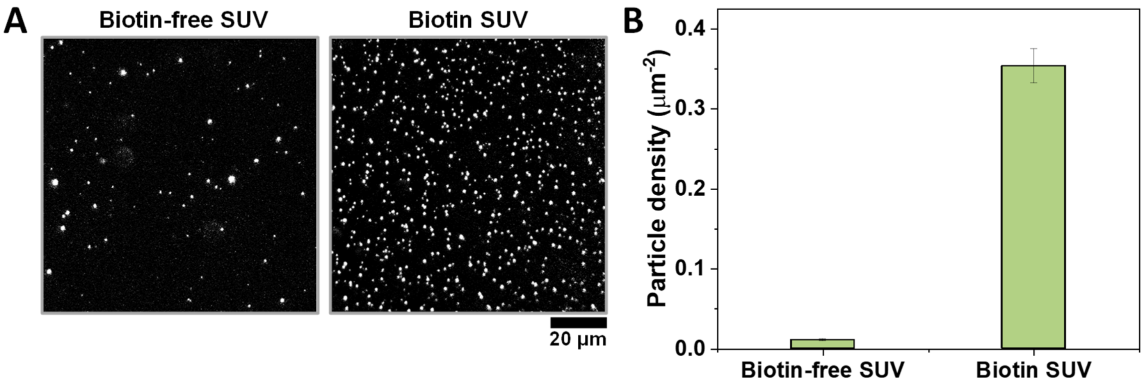

Figure S1. Biotin-free SUVs adsorb poorly to the functionalized surface. (A) Representative TIRF images of biotin-free SUVs and biotinylated SUVs on the functionalized surface. (B) Characterization of particle densities of biotin-free SUVs and biotinylated SUVs on the functionalized surface. Two individual imaging experiments were performed with two different batches of SUV samples. Four 100 µm x 100 µm regions were randomly selected for each sample, and all particles in these regions were sampled and analyzed. Error bars represent standard error among selected regions.

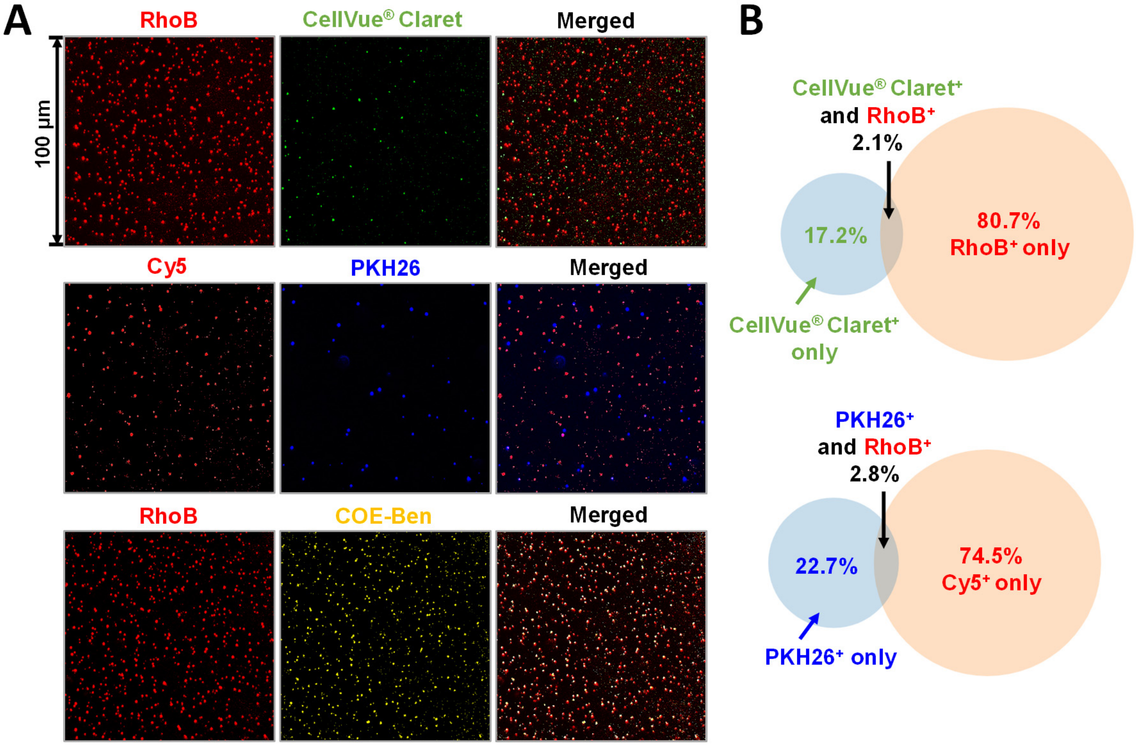

Figure S2. (A) TIRF images of SUVs stained with three different membrane probes. The SUVs contain fluorescent lipid of Rhodamine B-PE or Cy5-PE during the synthesis process. (B) Colocalization analysis of SUVs that are stained with both lipid-dye conjugates (RhoB-PE or Cy5-PE) and either of two commonly

used membrane probes (CellVue® Claret or PKH26). Three individual imaging experiments were performed with two different batches of SUV samples for both CellVue® Claret and PKH26. Eight 100 µm x 100 µm regions were randomly selected, and all particles in these regions were sampled and analyzed. As reported in our previous publications,[1,2] COE molecules can potentially have membrane disrupting properties. Therefore, we hypothesize that the COE-Ben-only population may contain COE-disrupted SUVs, where positively charged COE-Ben extracts negatively charged RhoB-PE and forms lipid nano-assemblies in the solution. Also, COE-Ben aggregates may contribute to this subpopulation. Note that such potential and moderate membrane-disruption effect of COE-Ben is not expected to destroy sEVs or alter sEVP subpopulation distributions. Firstly, the potential membrane-disruption effect of COE-Ben, at most, affected ~8.7 % of the total SUVs (Figure 2). Since sEVPs are generally more stable than SUVs, we expect that any such effect would impact fewer sEVPs than SUVs. Secondly, as demonstrated in Figure S18, increasing the COE-Ben concentration from 0.1 µM to 0.5 µM resulted in an increased detection of sEV population. As for the RhoB-only subpopulation shown in Figure 2, it could contain SUVs that either fail to incorporate COE-Ben or exhibit quenched COE-Ben intensity due to a FRET effect between COE-Ben and RhoB.

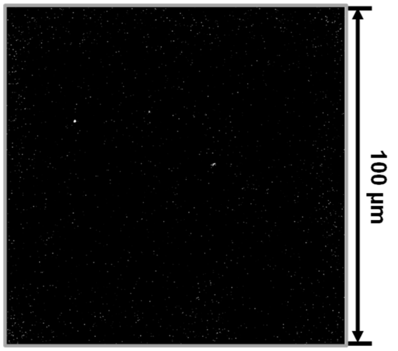

Figure S3. TIRF image of 0.5 µM COE-Ben on the functionalized surface without SUVs. The poor signal of COE-Ben can be due to both low nonspecific surface adsorption and a lack of membrane intercalation.[3] The lack of non-specific adsorption of COE-Ben to the surface can be explained by the high water solubility of COE-Ben and the electrostatic repulsion between positively charged poly(L-lysine) and positively charged COE-Ben.

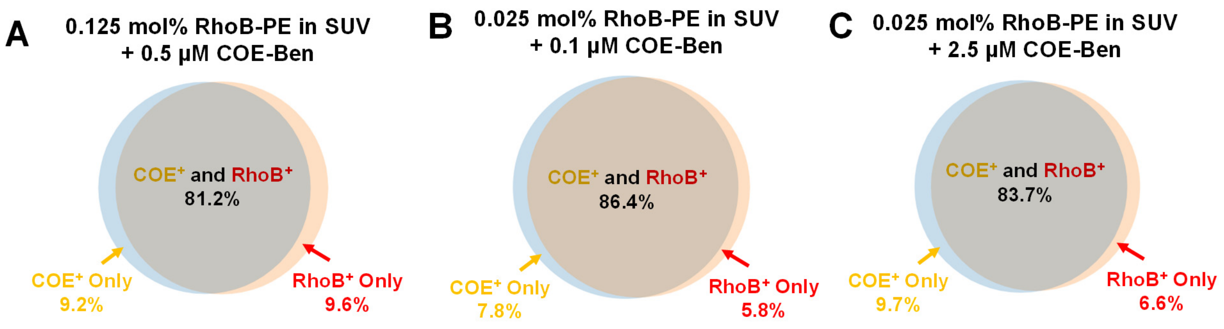

Figure S4. Colocalization analysis of RhoB and COE-Ben intensities per SUV in three different conditions: A) 0.125 mol% RhoB-PE in the SUVs with 0.5 μM COE-Ben; B) 0.025 mol% RhoB-PE in the SUVs with 0.1 μM COE-Ben; C) 0.025 mol% RhoB-PE in the SUVs with 2.5 μM COE-Ben. Two individual imaging experiments were performed with one batch of SUV sample for each condition. Four 100 μm x 100 μm regions were randomly selected for each sample, and all particles in these regions were sampled and analyzed.

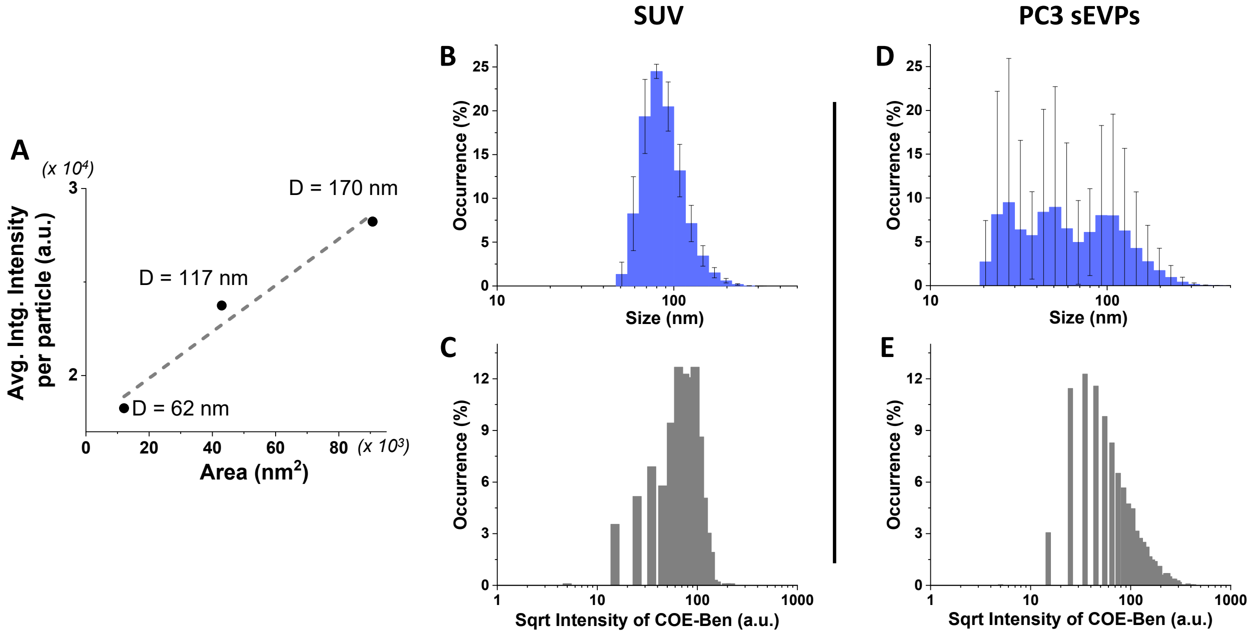

Figure S5. Characterizing COE-Ben intensity profile as a function of SUV size. (A) Average integrated COE-Ben intensity per SUV as a function of the average area of the SUVs determined by dynamic light scattering (DLS) measurement. To generate this plot, we first synthesized SUVs at three different sizes using extruder membranes with pore sizes of 30 nm, 100 nm, and 200 nm. The average diameters of the SUVs, measured via dynamic light scattering, were found to be 62 nm, 117 nm, and 170 nm, respectively. The SUVs were then stained with COE-Ben, following the protocol described in the Methods section of the main text. Next, we imaged the three SUV samples individually and analyzed their average integrated intensity of COE-Ben per particle. Two individual imaging experiments were performed with one batch of SUV sample for each size. Six 100 μm x 100 μm regions were randomly selected for each sample, and all particles in these regions were sampled and analyzed for the average integrated intensity per particle. (B)

Size distribution of SUVs (passed through a 100 nm membrane filter) as measured by DLS. Error bars represent standard deviation from two SUV samples. (C) Distribution of the square root of integrated COE-Ben intensity per SUV as imaged by TIRF. Two individual imaging experiments were performed with two batches of SUV samples. Six 100 µm x 100 µm regions were randomly selected, and all particles in these regions were sampled and analyzed. The consistency of these two distributions in (B) and (C) suggests that the fluorescence intensity of a COE-stained SUV is proportional to the surface area of the lipid membrane. (D) Size distribution of PC3 sEVPs as measured by DLS. Error bars represent standard deviation from two batches of sEVP samples. (E) Distribution of the square root of integrated COE-Ben intensity per sEVP as imaged by TIRF. Two individual imaging experiments were performed with two batches of sEVP samples. Five 100 µm x 100 µm regions were randomly selected, and all particles in these regions were sampled and analyzed. Given that there is a significant amount of non-vesicular particles that cannot be stained by COE-Ben within the sEVP sample, the two distribution profiles in (D) and (E) do not match each other.

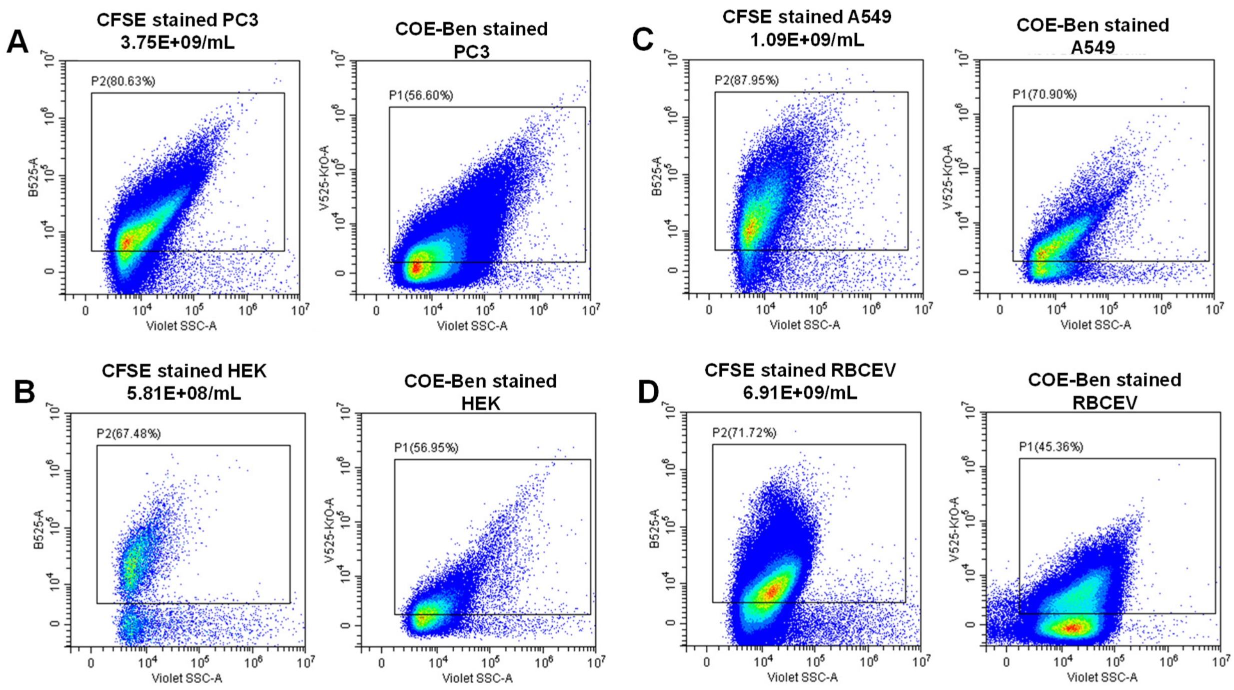

Figure S6. Staining ratio of CFSE and COE-Ben characterized using flow cytometry. Flow cytometry data were collected on a CytoFLEX LX (Beckman Coulter) based on sEVPs ((A) PC3, B) HEK293, C) A549, D) RBC) stained either by 5 µM CFSE or 0.5 µM COE-Ben in 1x PBS. The staining ratios (positive event rates) of both dyes are displayed in the figures, and total particle numbers are displayed in the figure captions. These measurements were taken with CytoFLEX instead of NanoFCM because NanoFCM was not able to detect signals from COE-Ben due to limitation in excitation wavelengths. Capture fractions (θ) are calculated following the equation: $\theta = (\rho A))/((NV)$, where ρ is the surface density of captured particles, A is the total surface area of the imaging chamber (0.6 cm^2), N is the bulk particle concentration as measured by flow cytometry, and V is the total volume of the imaging chamber (30 µL).

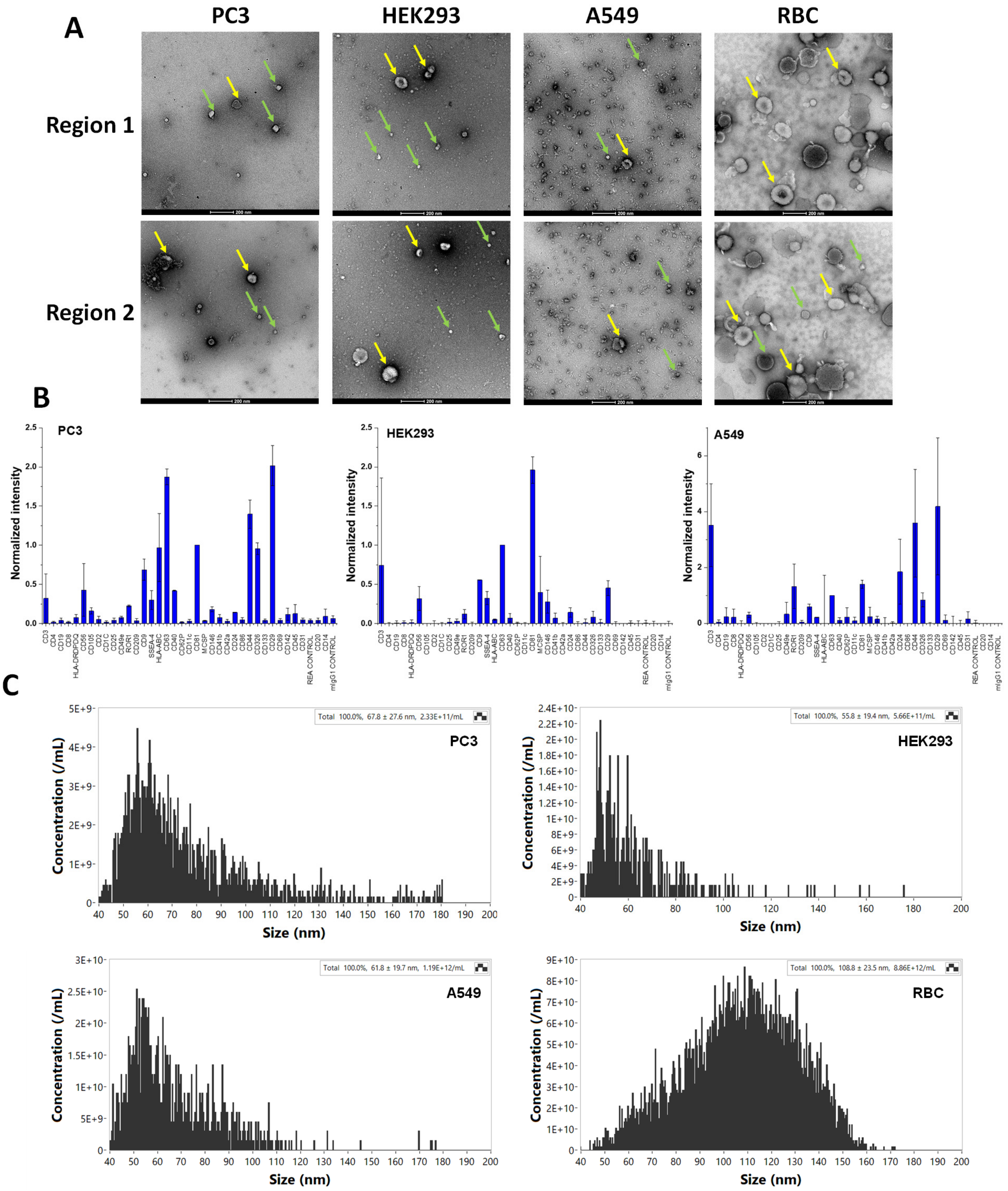

Figure S7. Verification of the quality of purified sEVPs. (A) Negative-stain TEM images of sEVPs showing vesicular (yellow arrows) and non-vesicular particles (green arrows). Specifically, sEVs are cup-shaped with membranes deformed due to dehydration, and they are usually larger than NPs.[4-6] At least two different batches of samples for each sEVP type were imaged, and no noticeable discrepancy was observed during imaging between the two batches. (B) Characterization of EV surface markers on sEVPs (PC3, HEK, A549) using flow cytometry. The intensities were obtained using the Miltenyi MACSPlex EV kit. Intensities of all

other markers are normalized to either CD63 or CD81. Thus, peak intensities are used to confirm the presence of specific markers on sEVPs and should not be used for direct, quantitative comparison of marker copy numbers across different types of sEVPs. Two individual measurements were performed with two batches of sEVP samples. Error bars represent standard error between the two trials. (C) Concentration and size distribution of sEVPs measured using nano-flow cytometry.

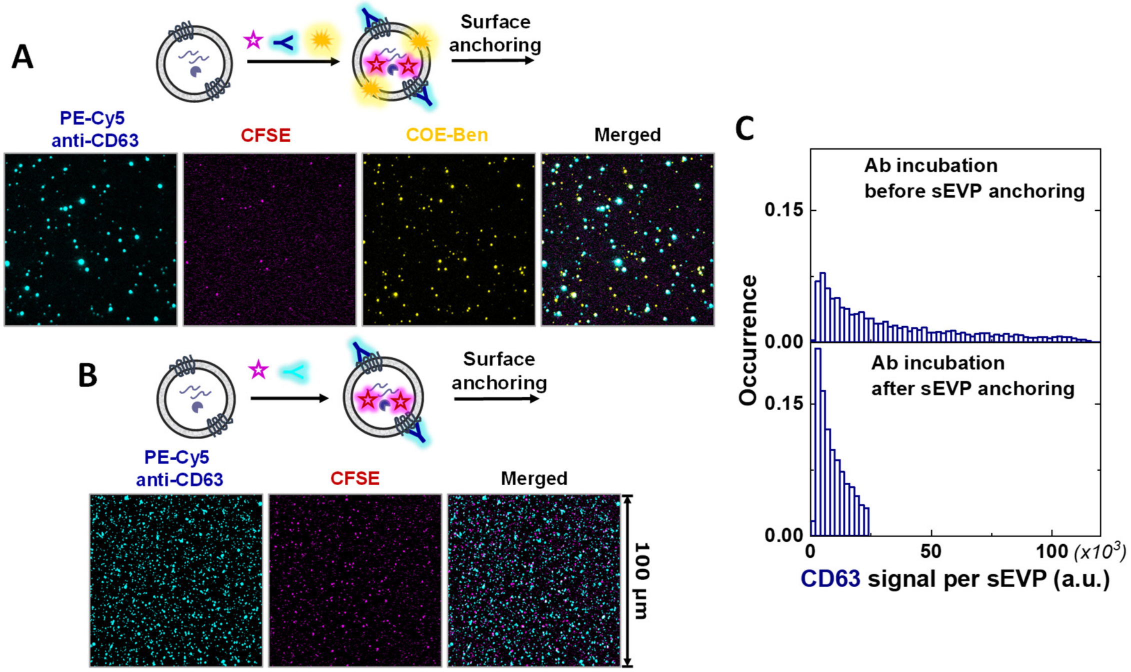

Figure S8. Optimization of a proper staining order for multidimensional fluorescent labeling. (A) When CD63-Abs, CFSE and COE-Ben were all co-incubated with sEVP samples before anchoring the particles onto a surface, there was a significant amount of aggregated sEVP particles. The positive charge of COE-Ben can decrease the negative surface charge on sEVPs, potentially reducing electrostatic repulsions among sEVPs. Therefore, it may become much easier for CD63-Abs to bridge between different sEVPs, ultimately leading to sEVP aggregation. (B) When CD63-Abs and CFSE were co-incubated with sEVP samples without COE-Ben before anchoring the particles onto a surface, the sEVPs remain well dispersed. (C) Incubating CD63-Abs with sEVPs before surface anchoring results in higher CD63 signal per particle than incubating them with surface-tethering sEVPs, potentially due to a steric effect. Together, results in this figure illustrate that antibody staining should happen before sEVP surface anchoring and COE-Ben staining should happen after surface anchoring. Two individual imaging experiments were performed with two batches of sEVP samples for each condition. Eight 100 μm x 100 μm regions were randomly selected, and all particles in these regions were sampled and analyzed.

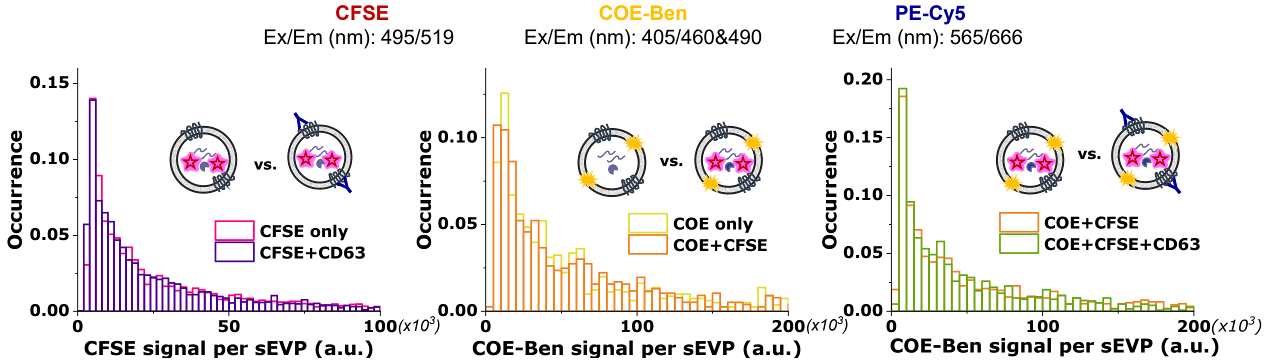

Figure S9. No detectable FRET effect among the three markers. Left panel: intensity histograms of CFSE signal per particle with and without PE-Cy5 labeled CD63 antibodies illustrate that there is negligible FRET effect between the CFSE and PE-Cy5 channels; middle panel: intensity histograms of COE-Ben per particle with and without CFSE illustrate that there is negligible FRET effect between the COE-Ben and CFSE channels; right panel: intensity histograms of COE-Ben signal per particle when stained with COE+CFSE or with COE+CFSE+CD63-Ab illustrate that there is negligible FRET effect between the COE-Ben and PE-Cy5 channels. Two individual imaging experiments were performed with two different batches of PC3 sEVP samples for each staining condition. For each condition, six 100 µm x 100 µm regions were randomly selected, and all particles in these regions were sampled and analyzed.

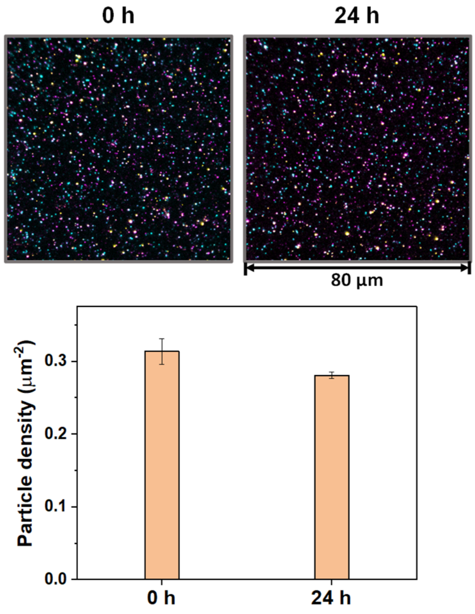

Figure S10. TIRF images of PC3 sEVPs at 0 h and 24 hours after anchoring to a NeuA surface. Both images were taken after washing the imaging chamber with copious buffer solution. Particle density at 0 h and 24 h was quantified. Two individual imaging experiments were performed with one batch of sEVP sample for each condition. Eight 100 µm x 100 µm regions were randomly selected, and all particles in these regions were sampled and analyzed. Error bars represent standard error among selected regions.

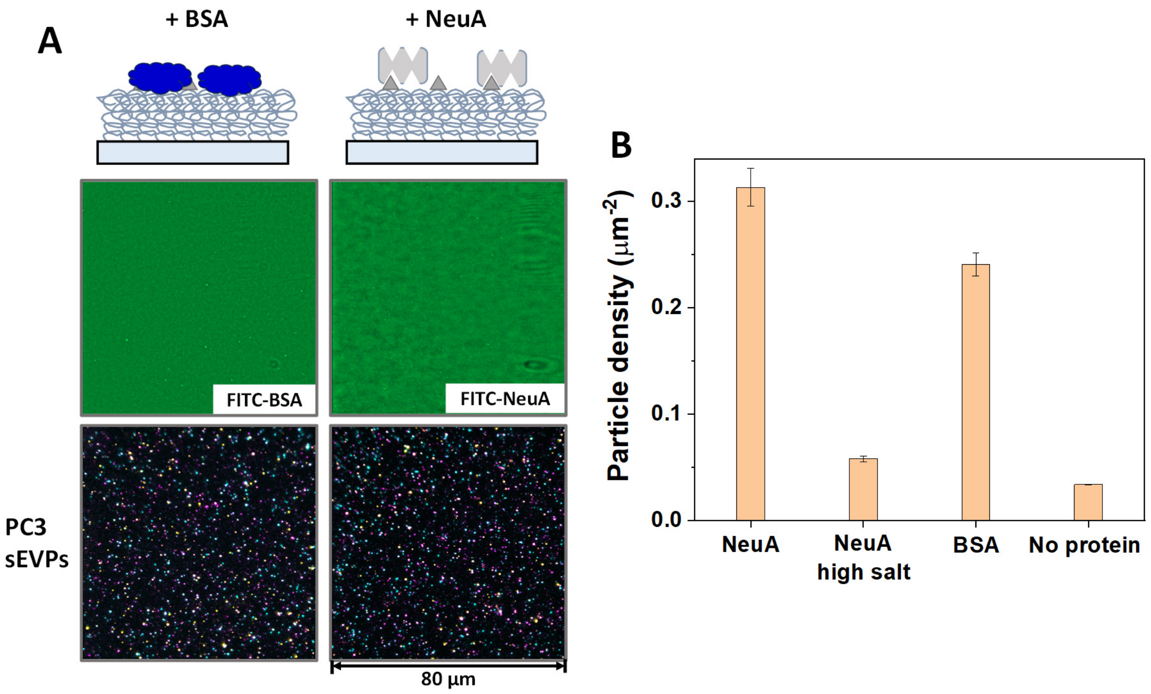

Figure S11. Characterizing surface attachment of sEVPs under different anchoring conditions. (A) TIRF images of fluorescein (FITC)-labeled BSA and NeuA forming uniform layers on PLL-PEG-biotin, as well as PC3 sEVPs captured on the BSA and NeuA protein layers. Electrostatic attractions between negatively charged BSA and positively charged PLL at the experimental pH can be the driving force for the formation of a BSA layer. (B) Particle density of captured sEVPs under different anchoring conditions: i) sEVPs incubated on the NeuA layer in 1x PBS, ii) sEVPs incubated on the NeuA layer in 1x PBS plus 300 mM NaCl, iii) sEVPs incubated on the BSA layer in 1x PBS, and iv) sEVPs incubated on the PLL-PEG-biotin layer directly without any anchoring protein. Increasing salt concentration led to a significant decrease of anchored particle density. This result indicates that, while the nonspecific interaction between sEVPs and anchoring proteins can have a complex nature, it involves electrostatic interactions that can be effectively screened with high concentration of buffer salt. For each condition, at least four 100 µm x 100 µm regions were randomly selected, and all particles in these regions were sampled to calculate the average particle density. Error bars represent standard error among selected regions.

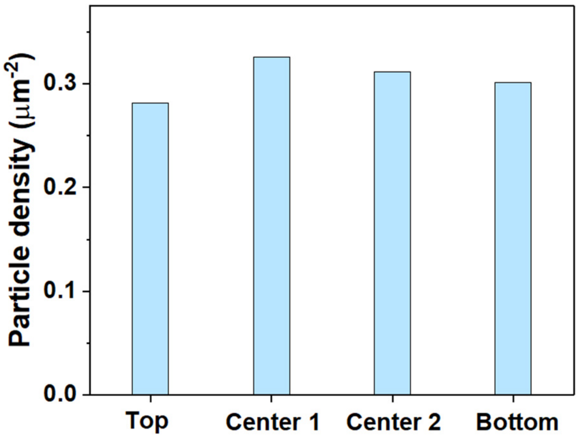

Figure S12. Particle density of PC3 sEVPs anchored on a NeuA layer, from the top to the center, and the bottom of an imaging chamber. On the 3.8 mm x 17 mm chip, the distance from the top to the center is approximately 8 mm, and from the center to the bottom is also approximately 8 mm. The two regions imaged at the center of the chip is approximately 1 mm apart horizontally.

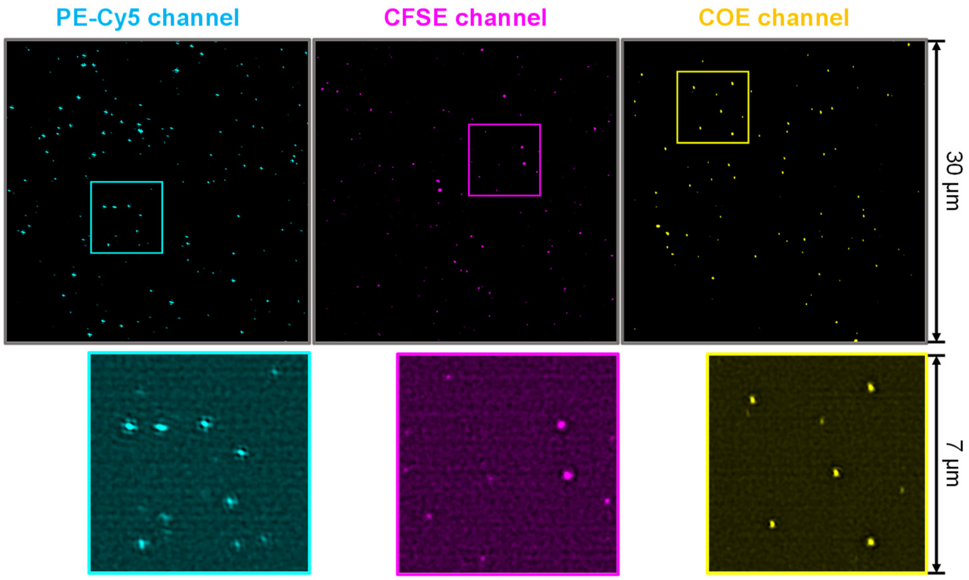

Figure S13. Super resolution structured illumination microscopy (SR-RIM) of surface-captured sEVPs in the PE-Cy5 channel, CFSE channel, and COE-Ben channel. At least two different batches of PC3 sEVP samples were imaged, and no noticeable discrepancy was observed during imaging between the two batches.

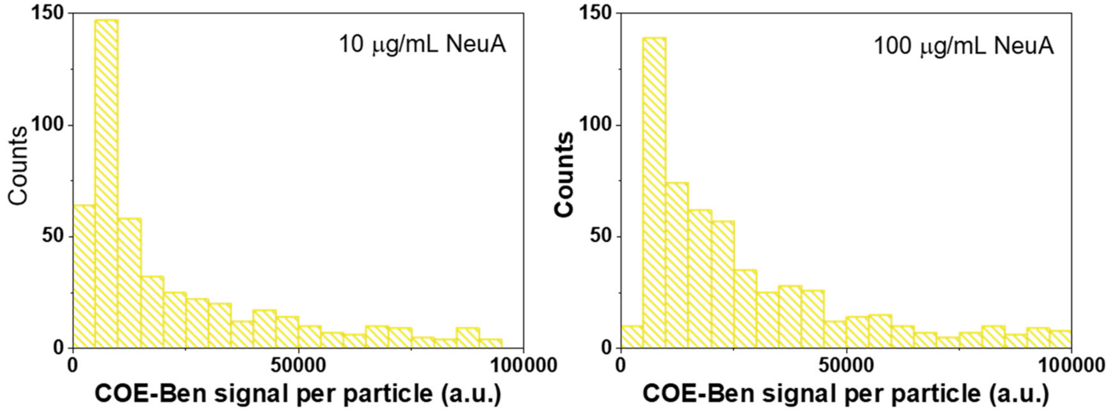

Figure S14. Intensity histograms of COE-Ben signal per particle when the coating protein NeuA concentration is at 10 μg/ml and 100 μg/ml, respectively. Two individual imaging experiments were performed with two different batches of PC3 sEVP samples for each NeuA concentration. At each NeuA concentration, six 100 μm x 100 μm regions were randomly selected, and all particles in these regions were sampled and analyzed.

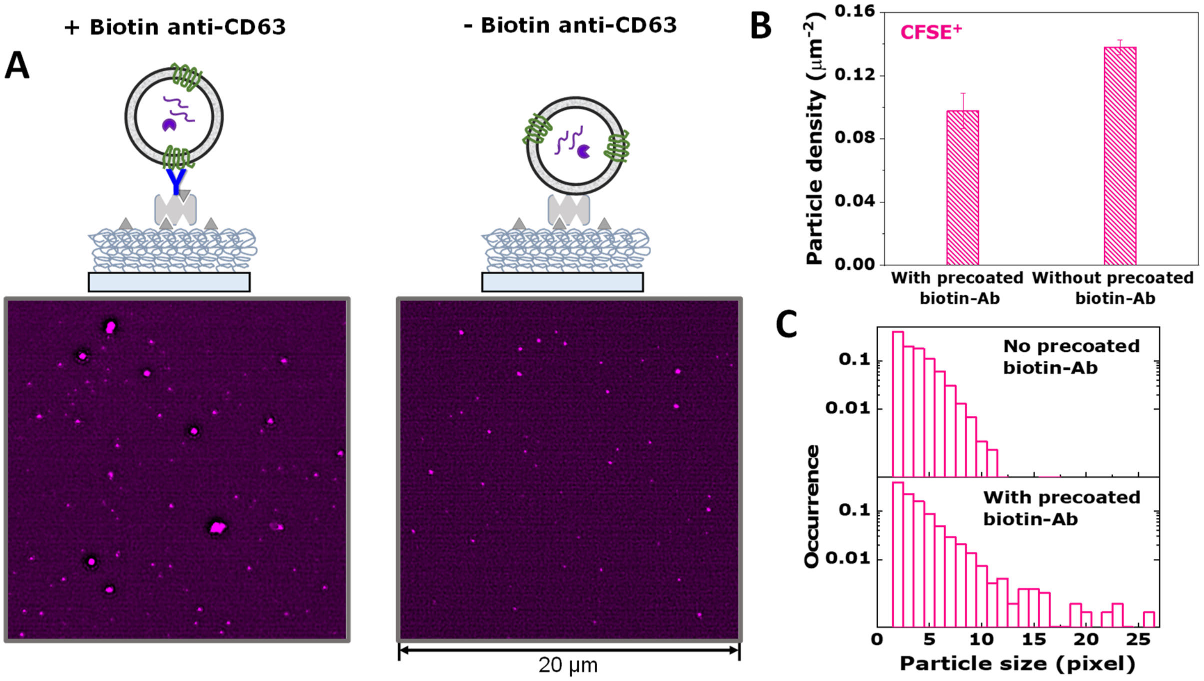

Figure S15. Comparison between sEVP-capturing setups with and without pre-coated antibodies. (A) Schematic demonstration and SR-SIM images of sEVP-capturing setups with and without pre-coated biotinylated CD63 antibodies (biotin-CD63 Abs). The PC3 sEVPs are stained with CFSE. It is possible that each NeuA binding site may capture more than one biotin-CD63 Abs, and the local high binding valency

from captured Abs may induce aggregation of sEVPs on the surface. Such aggregation can lead to a decreased apparent particle density. At least two different batches of PC3 sEVP samples were imaged, and no noticeable discrepancy was observed during imaging between the two batches. (B) Particle density, obtained by TIRF imaging, of the two sEVP-capturing schemes. (C) Particle size distribution, also obtained by TIRF imaging, with the two sEVP-capturing schemes. Two individual imaging experiments were performed with two different batches of PC3 sEVP samples for each condition. Under each condition, six 100 µm x 100 µm regions were randomly selected, and all particles in these regions were sampled and analyzed. Error bars represent standard error among selected regions.

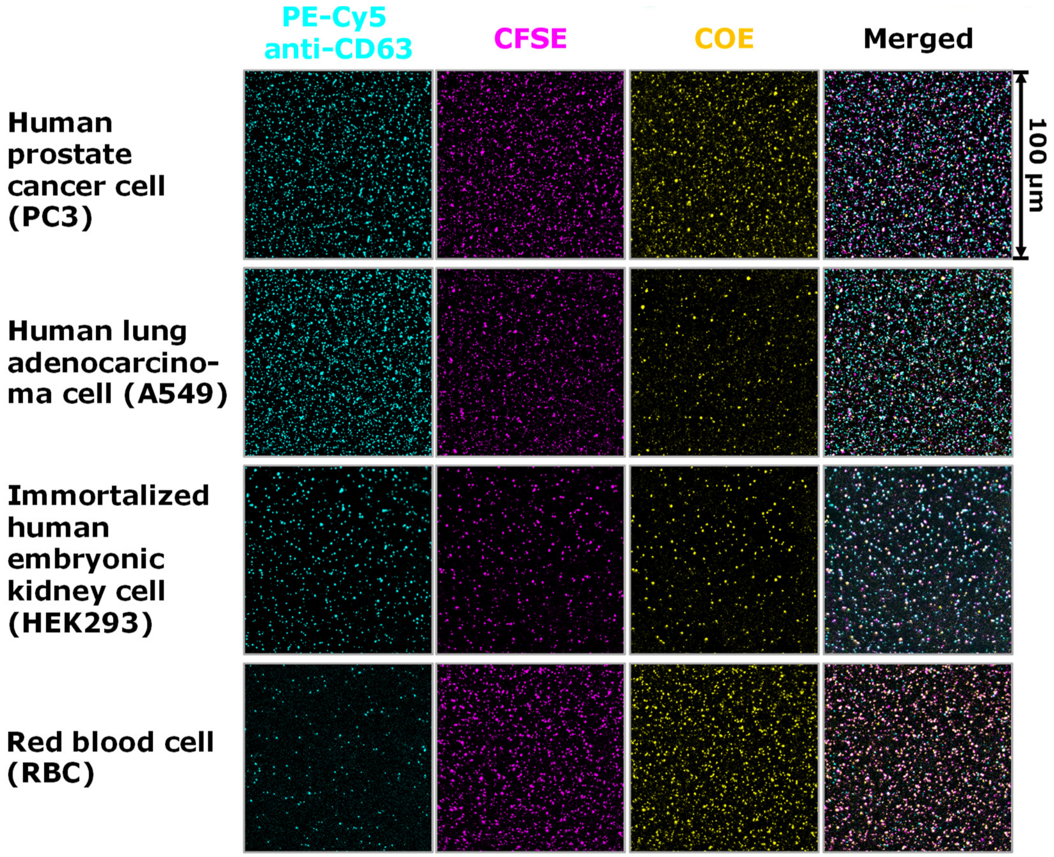

Figure S16. Representative TIRF images of four types of sEVPs in three fluorescence channels, presented as individual channels and as merged.

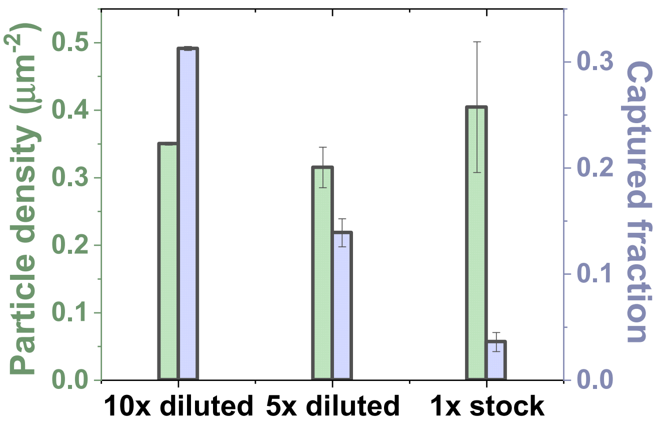

Figure S17. Surface densities and captured fractions of PC3 sEVPs at three different bulk sEVP concentrations: 10-times diluted, 5-times diluted, and undiluted from a stock concentration of 100 μg/mL. Two individual imaging experiments were performed with one batch of PC3 sEVP sample for each condition. Under each condition, at least six 100 μm x 100 μm regions were randomly selected, and all particles in these regions were sampled and analyzed. Error bars represent standard error among selected regions.

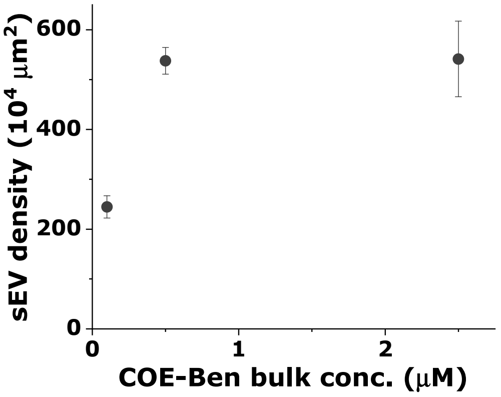

Figure S18. Number density of detectable sEVs as a function of COE-Ben bulk concentration during incubation. Two individual imaging experiments were performed with one batch of PC3 sEVP sample for each condition. Under each condition, at least four 100 μm x 100 μm regions were randomly selected, and all particles in these regions were sampled and analyzed. Error bars represent standard error among selected regions.

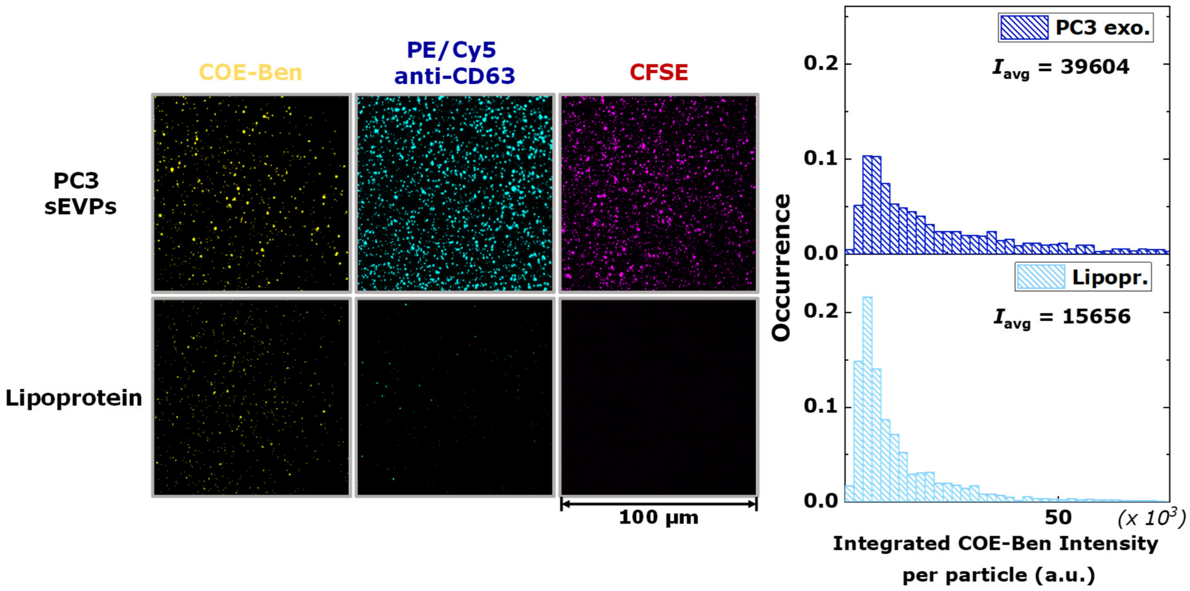

Figure S19. COE-Ben can stain lipoproteins. The average intensity of COE-Ben signal per lipoprotein is approximately half of that in individual PC3 sEVs. Given that the non-vesicular NPs identified in our assay cannot be stained by COE-Ben, we conclude that they are unlikely to be lipoproteins. Two individual imaging experiments were performed with one batch of PC3 sEVP and lipoprotein sample. At least four 100 µm x 100 µm regions were randomly selected, and all particles in these regions were sampled and analyzed.

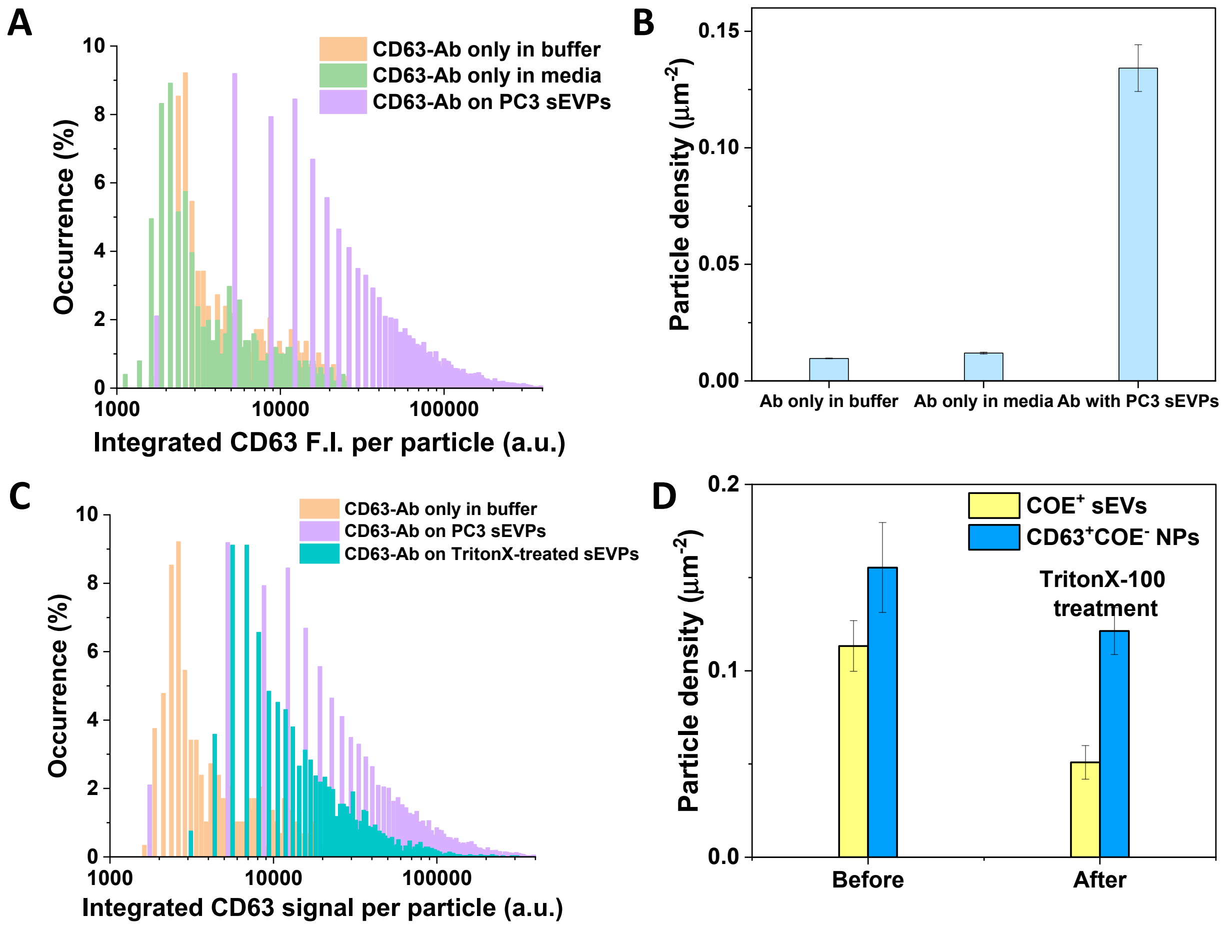

Figure S20. Characterizing $CD63^+$ NPs. (A) and (B): Intensity histograms and particle density of nonspecifically adsorbed CD63 antibodies in buffer solution and in cell culture medium. The intensity histogram and particle density of $CD63^+$ PC3 sEVPs are also included as a positive control. The consistency observed in buffer and cell culture medium conditions indicates that the cell culture medium did not contain or induce any protein aggregates that may contribute to the $CFSE^-CD63^+$ NP subpopulation. Two individual imaging experiments were performed with one batch of CD63 antibodies at each condition. At least four 100 μm x 100 μm regions were randomly selected for each condition, and all particles in these regions were sampled and analyzed. (C) Intensity histograms of: i) CD63 antibodies nonspecifically adsorbed, ii) CD63 antibodies on PC3 sEVPs, and iii) CD63 antibodies on Triton X-100-treated sEVPs. (D) Particle densities of COE^+ sEV and $CD63^+COE^-$ NPs before and after Triton X-100 treatment. To perform Triton X-100 treatment, 100% Triton X-100 stock solution (SigmaAldrich) was added to the sEVP sample at a final concentration of 1 %v/v. The mixture was then allowed to incubate at 37 °C for 30 min, with a 10 s vortex step every 10 min. Subsequently, the treated mixture was cleaned up using a 100 kDa Amicon Ultra-0.5 centrifugal filter unit. Such Triton X-100 treatment is expected to destabilize membrane structures and disrupt protein aggregates.[9,10] As illustrated in (D), while the majority of vesicular particles (COE^+) were disrupted by the surfactant (over 55% decrease in population), the density of non-vesicular $CD63^+$ particles decreased by less than 20%. Moreover, (C) demonstrates that Triton X-100 treatment shifted the intensity distribution of $CD63^+$ particles to moderately lower values; nevertheless, it remained notably higher than the intensity of single CD63 antibodies. Together, we conclude that the majority of non-vesicular $CD63^+$ particles are unlikely to be random protein aggregates.

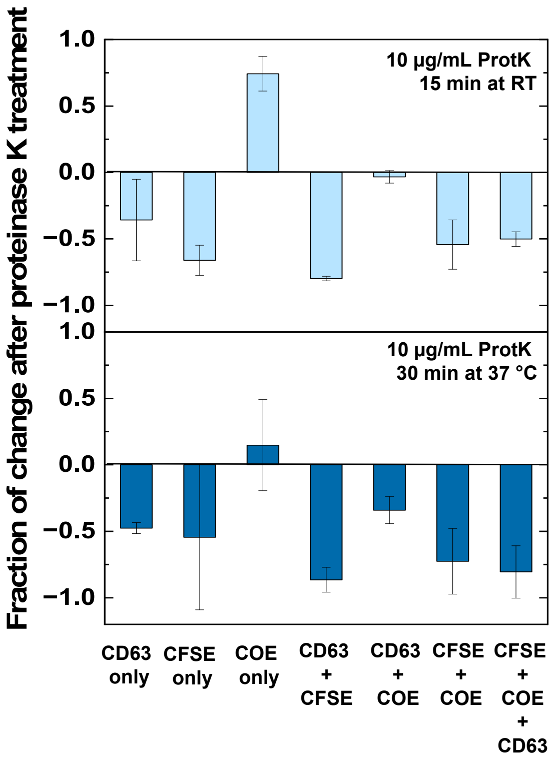

Figure S21. Change of particle number in each subpopulation after protease K treatment. Proteinase K was added to the sEVP samples at a final concentration of 0.01 mg/mL. The mixture was then allowed to incubate either at 37 °C for 30 min or at room temperature (24 °C ± 1 °C) for 15 min. Subsequently, the digested mixture was cleaned up using a 100 kDa Amicon Ultra-0.5 centrifugal filter unit. Under the milder treatment condition (15 min at RT), proteinase K preferentially digested COE^- particles, suggesting the protective effect of the membrane structure. Under the more intense treatment condition, COE^+ particles were also significantly disrupted, indicating that proteinase K digestion of sEVPs can destabilize the membrane structures. The proteinase K treatment is expected to digest the majority of surface proteins on sEVPs, as well as any potential protein aggregates in the samples. Additionally, this step can potentially reduce the binding affinity of sEVPs to NeuA. Therefore, the observed population decrease in variedsEVP subtypes can be due to both particle degradation and reduced affinity to NeuA. Regarding the COE^+-only subtypesubtype, it may be more resistant to proteinase K digestion due to a lower surface protein density. Moreover, digested products of the other six subtypes may contribute to this population. Therefore, we observed a consistentpopulation increase in this subtype. At least two individual imaging experiments were performed with two batch of samples at each condition. Four to six 100 μm x 100 μm regions were randomly selected for each condition, and all particles in these regions were sampled and analyzed. Error bars represent standard error among selected regions.

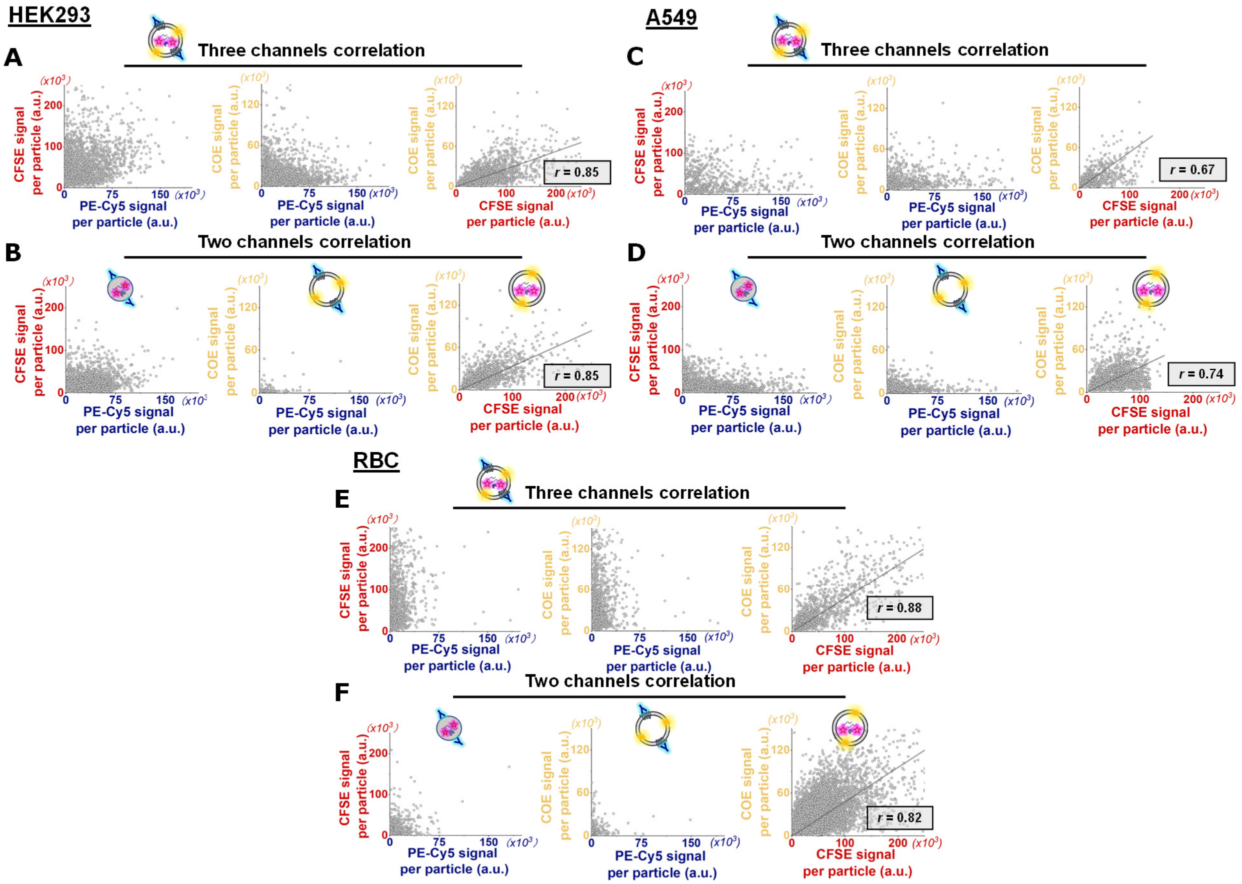

Figure S22. Two-dimensional intensity scatter plots of the three fluorescent labels on individual sEVPs from different subpopulations of sEVPs from HEK293, A549 and RBC. (A) Intensity scatter plots between each two of the three colocalized fluorescent markers in CFSE$^+$CD63$^+$ sEVs of HEK (include 1979 particles from three representative trials). (B) Intensity scatter plots between two colocalized fluorescent markers in CFSE$^+$CD63$^+$ NPs, CFSE$^-$CD63$^+$ sEVs, and CFSE$^+$CD63$^-$ sEVs of HEK, respectively (include 3746, 96, and 1036 particles from two representative trials, respectively). (C) Intensity scatter plots between each two of the three colocalized fluorescent markers in CFSE$^+$CD63$^+$ sEVs of A549 (include 670 particles from two representative trials). (D) Intensity scatter plots between two colocalized fluorescent markers in CFSE$^+$CD63$^+$ NPs, CFSE$^-$CD63$^+$ sEVs, and CFSE$^+$CD63$^-$ sEVs of A549, respectively (include 4013, 466, and 1481 particles from two representative trials, respectively). (E) Intensity scatter plots between each two of the three colocalized fluorescent markers in CFSE$^+$CD63$^+$ sEVs of RBC (include 1177 particles from three representative trials). (F) Intensity scatter plots between two colocalized fluorescent markers in CFSE$^+$CD63$^+$ NPs, CFSE$^-$CD63$^+$ sEVs, and CFSE$^+$CD63$^-$ sEVs of RBC, respectively (include 553, 219, and 11950 particles from three representative trials, respectively).

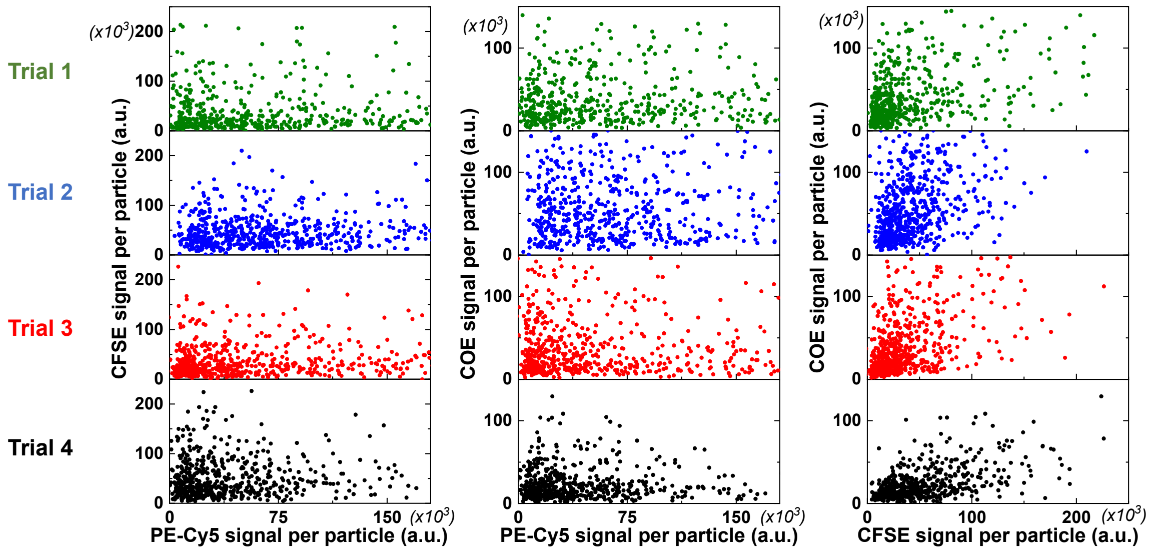

Figure S23. Intensity scatter plots between each two of the three colocalized fluorescent markers in CFSE$^+$ CD63$^+$ sEVs. Results from four different trials with three different batches of sEVP samples are included.

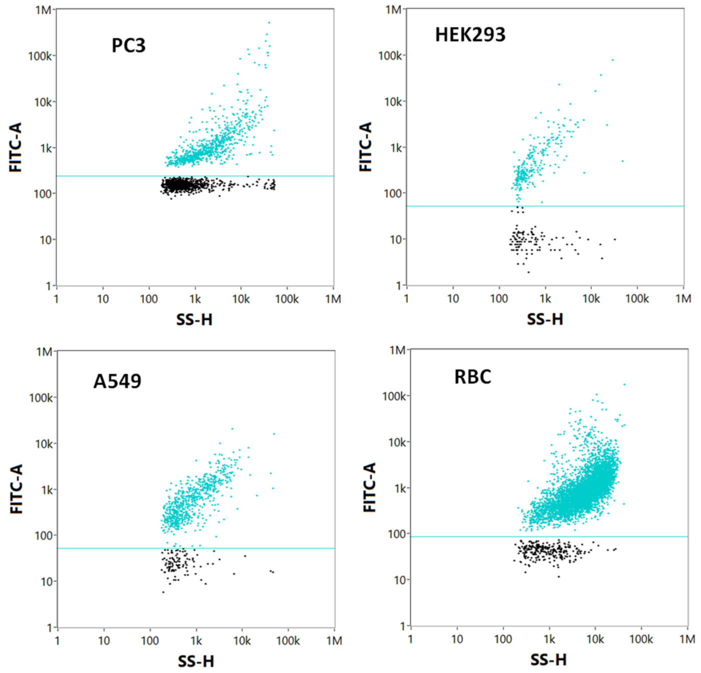

Figure S24. Characterization of CFSE signal as a function of particle size using flow cytometry. Flow cytometry data were collected on a NanoAnalyzer (NanoFCM) based on four different types of sEVPs stained by 5 μM CFSE in PBS. The particle size was detected by the scattering channel (x-axis, SS-H), and the CFSE intensity was detected in the FITC channel (y-axis, FITC-A).

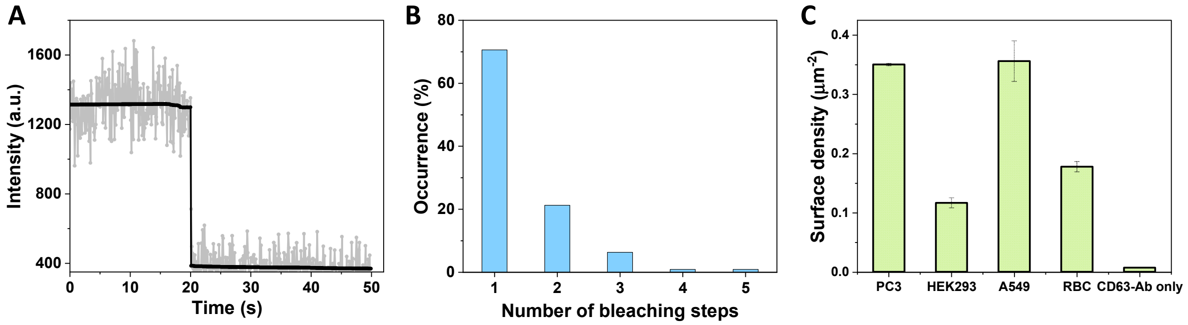

Figure S25. Profiling non-specifically adsorbed CD63 Abs on the NeuA-functionalized surface without sEVPs. (A) A representative single-step bleaching curve of CD63 Ab monomers nonspecifically adsorbed to the functionalized surface. PE-Cy5 labeled CD63 Abs (400 times diluted from a 200 μg/mL stock) were first centrifuged at 9000 RCF for 15 min at 4 °C, and 100 μL supernatant was introduced to incubate with the functionalized surface for 15 min in the absence of sEVPs. Then the imaging chamber was washed with copious buffer to remove unattached Abs. To gradually bleach the nonspecifically adsorbed antibodies, livestream videos were taken by TIRF microscopy with 500 frames at a framerate of 10 s^{-1}. (B) Histogram of bleaching steps of over 200 CD63 Abs nonspecifically deposited on the NeuA-functionalized surface. To determine the bleaching steps of individual Abs, the bleaching profiles all CD63 Ab molecules were analyzed using a previously published Bayesian change point detection and time series decomposition algorithm[7] in MATLAB. The number of bleaching steps automatically identified by the algorithm was confirmed manually, ensuring that dye blinking events did not interfere with bleaching step counts. We find that the majority of individual CD63 Abs demonstrated a single-step bleaching profile. This result is consistent with the conjugation ratio of dye to antibody provided by the vendor (Biolegend, lot# B342728), which is 1.23. Therefore, we are able to resolve the signal from a single fluorophore, and that the majority of antibodies adsorbed are in monomeric form. (C) Characterizing the surface density of non-specifically deposited CD63 Abs, in comparison with surface densities of attached sEVPs.

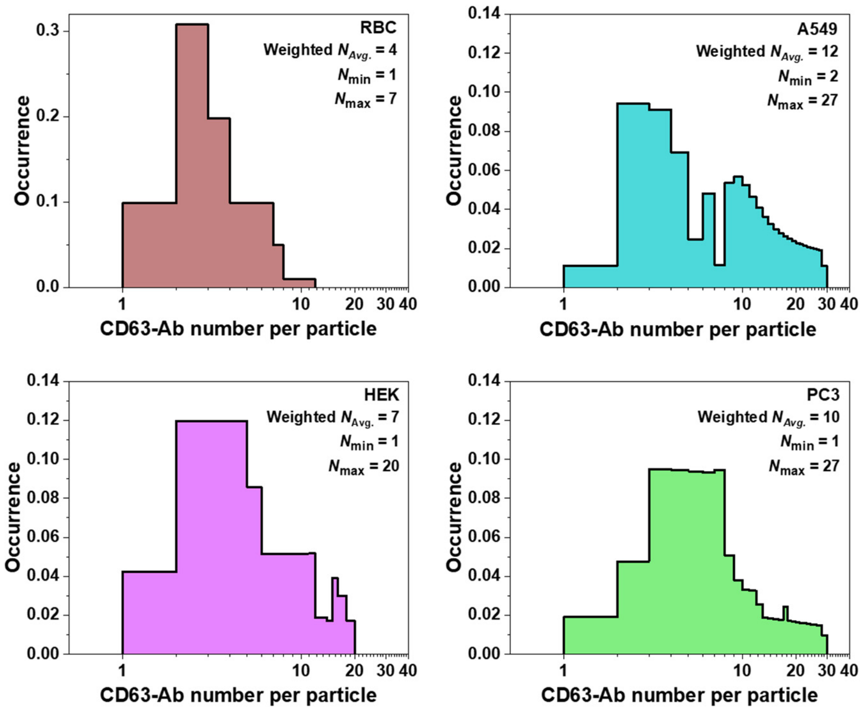

Figure S26. Distribution of CD63 counts on individual CFSE$^+$CD63$^+$ sEVs from four different cell types as calculated using a previously published intensity distribution deconvolution method.[8] Single-Ab intensity distribution was first scaled by integer values (1 through N) to create theoretical distributions for N number of Abs. The best fit of a linear combination of the theoretical distributions to the measured intensity distribution of CD63$^+$ labeled sEVPs was obtained using the least-squares solver in MATLAB. From the fit, both the weighted average number and variation in the number of CD63 per particle can be obtained. Note that only the N values with a weight larger than 1% are included.

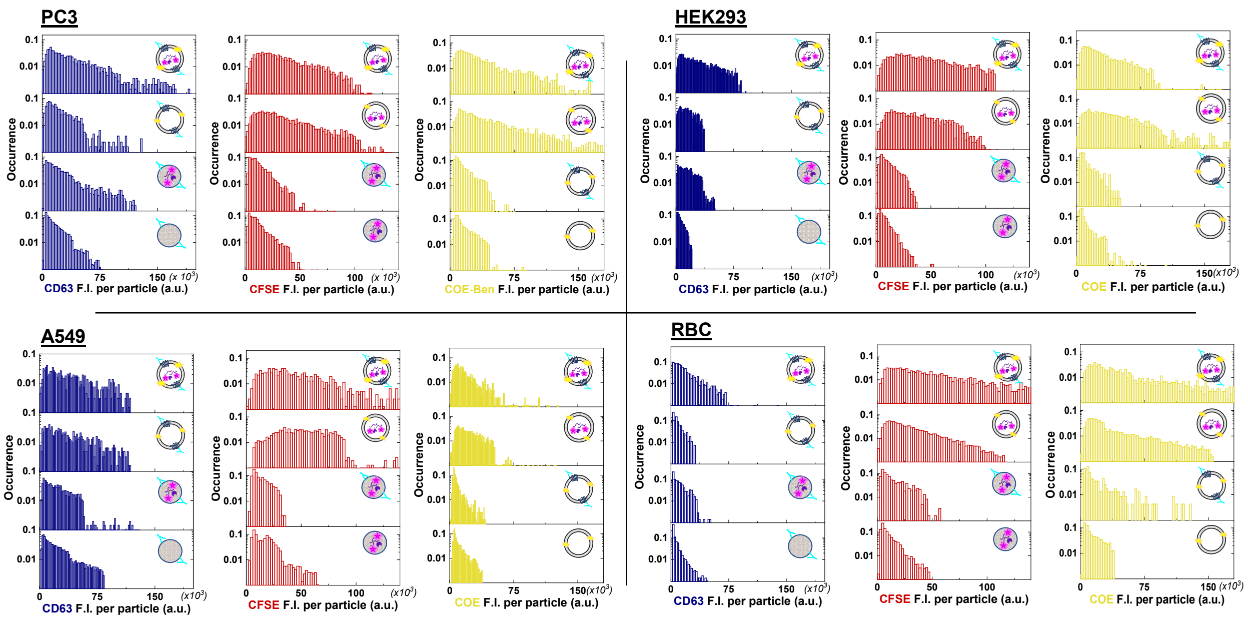

Figure S27. Intensity histograms of the three markers on individual particles in different subpopulations of sEVPs from PC3, HEK293, A549 and RBC, respectively. At least three individual imaging experiments were performed with three batches of PC3, HEK, A549, and RBC sEVP samples. For each sample, ten to fifteen 100 µm x 100 µm regions were randomly selected, and all particles (31380 particles for PC3, 21200 particles for HEK, 23411 particles for A549, and 20183 particles for RBC) in these regions were sampled and analyzed.

Table S1. Summary of subpopulation distributions in four different types of sEVP samples. Standard deviations were calculated from at least three individual experiments.

	**CFSE^{+} COE^{+} CD63^{+}**	**CFSE^{+} COE^{+} CD63^{-}**	**CFSE^{-} COE^{+} CD63^{+}**	**CFSE^{-} COE^{+} CD63^{-}**	**CFSE^{+} COE^{-} CD63^{+}**	**CFSE^{+} COE^{-} CD63^{-}**	**CFSE^{-} COE^{-} CD63^{+}**
**PC3** (31380 particles in total)	10.6 ± 1.6 %	11.9 ± 2.2 %	4.4 ± 2.4 %	10.4 ± 8.1 %	15.0 ± 6.9 %	12.9 ± 5.4 %	34.8 ± 1.1 %
**HEK293** (21200 particles in total)	14.9 ± 7.1 %	12.0 ± 8.9 %	2.6 ± 2.5 %	6.7 ± 6.0 %	20.9 ± 15.0 %	12.2 ± 7.9 %	30.7 ± 3.9 %

**A549** (23411 particles in total)	3.0 ± 2.0 %	7.8 ± 6.4 %	2.5 ± 0.8 %	7.1 ± 6.0 %	10.2 ± 9.8 %	11.5 ± 3.5 %	58.0 ± 2.6 %
**RBC** (20183 particles in total)	4.3 ± 1.4 %	40.5 ± 9.6 %	1.1 ± 0.5 %	19.1 ± 9.4 %	2.3 ± 0.4 %	19.3 ± 7.5 %	13.4 ± 9.1 %

References

[1] Yan, H.; Rengert, Z. D.; Thomas, A. W.; Rehermann, C.; Hinks, J.; Bazan, G. C. Influence of molecular structure on the antimicrobial function of phenylenevinylene conjugated oligoelectrolytes. *Chem. Sci.* **2016**, *7* (9), 5714–5722.

[2] Chan, S. J. W.; Zhang, K.; Zhu, J.; Bazan, G. C. Antimicrobial conjugated oligoelectrolytes containing triphenylphosphonium solubilizing groups. *Chemistry A European J.* **2023**, *29* (26), e202203803.

[3] Zhou, C.; Cox-Vázquez, S. J.; Chia, G. W. N.; Vázquez, R. J.; Lai, H. Y.; Chan, S. J. W.; Limwongyut, J.; Bazan, G. C. Water-Soluble Extracellular Vesicle Probes Based on Conjugated Oligoelectrolytes. *Sci. Adv.* **2023**, *9* (2), eade2996.

[4] Welsh, J. A.; Goberdhan, D. C. I.; O'Driscoll, L.; Buzas, E. I.; Blenkiron, C.; et al. Minimal Information for Studies of Extracellular Vesicles (MISEV2023): From Basic to Advanced Approaches. *J. of Extracellular Vesicle* **2024**, *13* (2), e12404.

[5] Théry, C.; Amigorena, S.; Raposo, G.; Clayton, A. Isolation and Characterization of Exosomes from Cell Culture Supernatants and Biological Fluids. *Curr. Protoc. Cell Biol.* **2006***, Chapter 3, Unit 3.22.*

[6] Zhang, H.; Freitas, D.; Kim, H. S.; Fabijanic, K.; Li, Z.; et al. Identification of Distinct Nanoparticles and Subsets of Extracellular Vesicles by Asymmetric Flow Field-Flow Fractionation. *Nat. Cell Biol.* **2018**, *20* (3), 332–343.

[7] Zhao, K.; Wulder, M. A.; Hu, T.; Bright, R.; Wu, Q.; et al. Detecting Change-Point, Trend, And Seasonality in Satellite Time Series Data To Track Abrupt Changes And Nonlinear Dynamics: A Bayesian Ensemble Algorithm. *Remote Sens. Environ.* **2019**, *232*, 111181.

[8] Mutch, S. A.; Gadd, J. C.; Fujimoto, B. S.; Kensel-Hammes, P.; Schiro, P. G.; Bajjalieh, S. M.; Chiu, D. T. Determining the Number of Specific Proteins in Cellular Compartments by Quantitative Microscopy. *Nat. Protoc.* **2011**, *6*, 1953–1968.

[9] Koley, D.; Bard, A. J. Triton X-100 Concentration Effects on Membrane Permeability of a Single HeLa Cell by Scanning Electrochemical Microscopy (SECM). *Proc. Natl. Acad. Sci. U.S.A.* **2010**, *107*, 16783–16787.

[10] Royster, A.; Mir, S.; Mir, M. A. A Novel Approach for the Purification of Aggregation Prone Proteins. *PLoS ONE* **2021**, *16*, e0260143.
